# Nitric oxide mediates red light-induced perylenequinone production in *Shiraia* mycelium culture

**DOI:** 10.1186/s40643-023-00725-5

**Published:** 2024-01-02

**Authors:** Wen Juan Wang, Xin Ping Li, Wen Hao Shen, Qun Yan Huang, Rui Peng Cong, Li Ping Zheng, Jian Wen Wang

**Affiliations:** 1 College of Pharmaceutical Sciences, Soochow University, Suzhou, 215123 China; 2 Department of Horticultural Sciences, Soochow University, Suzhou, 215123 China

**Keywords:** *Shiraia*, Red light, Nitric oxide, Perylenequinone, Biosynthesis, Elicitation

## Abstract

Perylenequinones (PQs) from bambusicolous *Shiraia* fungi serve as excellent photosensitizers for photodynamic therapy. However, the lower yield of PQ production in mycelium cultures is an important bottleneck for their clinical application. Light has long been recognized as a pivotal regulatory signal for fungal secondary metabolite biosynthesis. In this study, we explored the role of nitric oxide (NO) in the growth and PQ biosynthesis in mycelium cultures of *Shiraia* sp. S9 exposed to red light. The continuous irradiation with red light (627 nm, 200 lx) suppressed fungal conidiation, promoted hyphal branching, and elicited a notable increase in PQ accumulation. Red light exposure induced NO generation, peaking to 81.7 μmol/g FW on day 8 of the culture, with the involvement of nitric oxide synthase (NOS)- or nitrate reductase (NR)-dependent pathways. The application of a NO donor sodium nitroprusside (SNP) restored conidiation of *Shiraia* sp. S9 under red light and stimulated PQ production, which was mitigated upon the introduction of NO scavenger carboxy-PTIO or soluble guanylate cyclase inhibitor NS-2028. These results showed that red light-induced NO, as a signaling molecule, was involved in the regulation of growth and PQ production in *Shiraia* sp. S9 through the NO-cGMP-PKG signaling pathway. While mycelial H_2_O_2_ content exhibited no significant alternations, a transient increase of intracellular Ca^2+^ and extracellular ATP (eATP) content was detected upon exposure to red light. The generation of NO was found to be interdependent on cytosolic Ca^2+^ and eATP concentration. These signal molecules cooperated synergistically to enhance membrane permeability and elevate the transcript levels of PQ biosynthetic genes in *Shiraia* sp. S9. Notably, the combined treatment of red light with 5 μM SNP yielded a synergistic effect, resulting in a substantially higher level of hypocrellin A (HA, 254 mg/L), about 3.0-fold over the dark control. Our findings provide valuable insights into the regulation of NO on fungal secondary metabolite biosynthesis and present a promising strategy involving the combined elicitation with SNP for enhanced production of photoactive PQs and other valuable secondary metabolites in fungi.

**Graphical Abstract:**

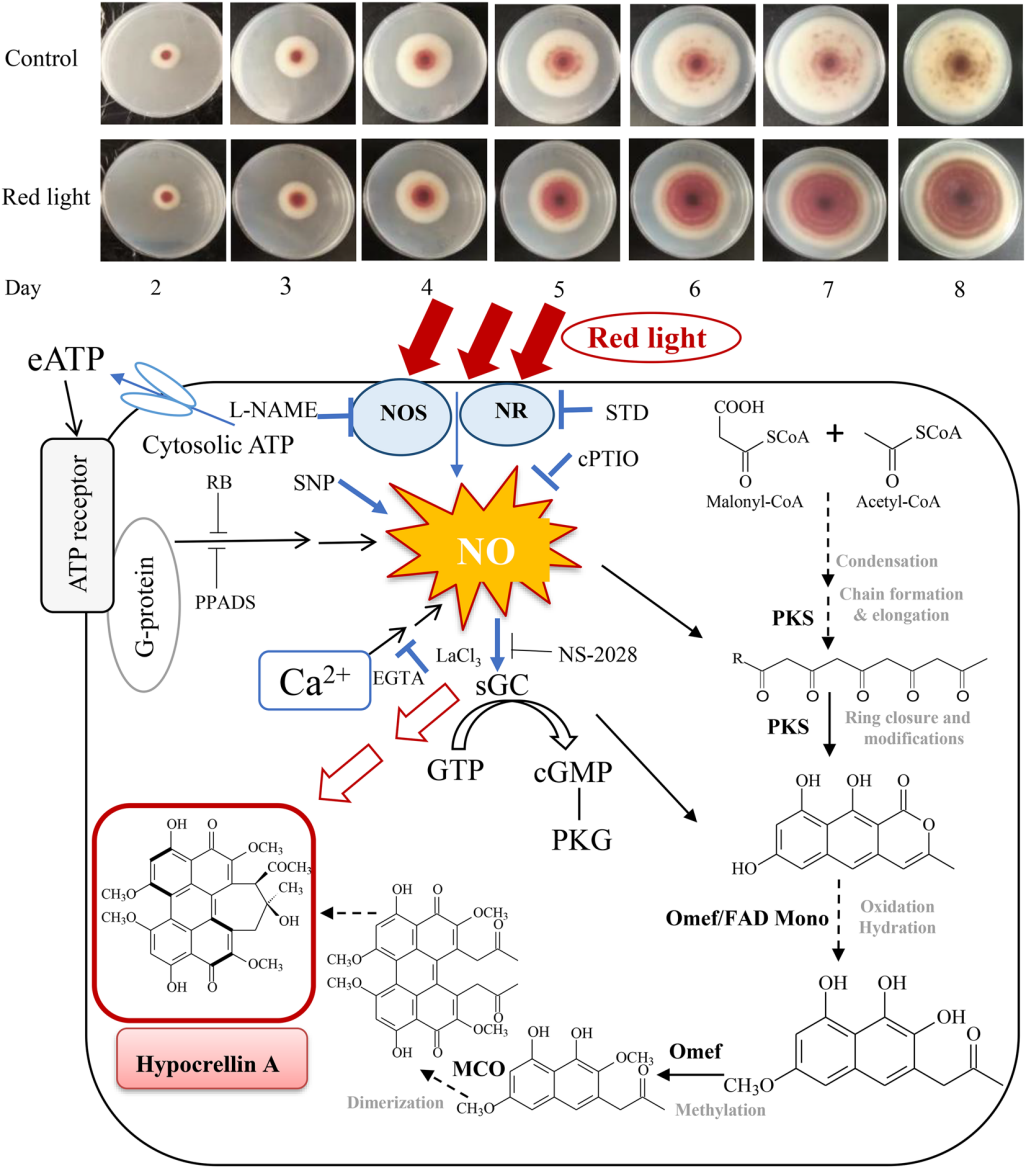

**Supplementary Information:**

The online version contains supplementary material available at 10.1186/s40643-023-00725-5.

## Introduction

*Shiraia bambusicola* is a pathogenic fungus to parasitize bamboo twigs, and its fruiting body has been used traditionally in Chinese medicine as Zhu Huang to treat conditions like rheumatoid arthritis, tracheitis and pains (Zhong and Xiao 2009). *Shiraia* hypha and fruiting bodies contain bioactive perylenequinones (PQs) such as hypocrellins and elsinochromes (Khiralla et al. 2022). Hypocrellin A (HA), a type of hypocrellins, exhibits strong photodynamic effects on tumor cells and microbial pathogens when exposed to visible light and oxygen. It has advantages such as high yields of reactive oxygen species (ROS), low dark toxicity, and rapid metabolism for the biomedical application (Miller et al. 1997). However, due to difficulties in artificial cultivation of the fruiting body and challenges in chemical synthesis of PQs like hypocrellins, *Shiraia* mycelium cultures have emerged as a biotechnological alternative for PQ production (Zhao and Liang 2005; Yang et al. 2009). To improve the low PQ productivity in *Shiraia* mycelium cultures, various abiotic elicitation strategies have been explored, including ultrasound exposure (Sun et al. 2017), Triton X-100 treatment (Lei et al. 2017) and lanthanum elicitation (Lu et al. 2019). Light, as an abiotic factor for *Shiraia*, also plays a role in regulating hypocrellin biosynthesis. For instance, Gao et al. (2018) reported that different light conditions promoted *S. bambusicola* growth, but decreased hypocrellin production. Our previous research showed that a light/dark shift (24 h: 24 h) stimulated HA production in *Shiraia* sp. S8 (Sun et al. 2018). Recent findings indicated that continuous blue-light exposure inhibited *Shiraia* HA production (Li et al. 2022), while red light (627 nm) at 200 lx significantly promoted HA (Ma et al. 2019a). Red light has been recognized as an essential environmental signal that facilitates the production of fungal metabolites, such as exo- and endo- polysaccharides of *Ganoderma lucidum* (Poyedinok et al. 2008), mycophenolic acid of *Penicillium brevicompactum* (Shu et al. 2010), and fumonisin B1-B3 of *Fusarium verticillioides* (Fanelli et al. 2012).

Following light perception, small molecular signals such as reactive oxygen species (ROS), adenosine triphosphate (ATP) and ion fluxes are induced to amplify the light signal for downstream reactions, including secondary metabolite production (Tisch and Schmoll 2010). For example, red light significantly promoted H_2_O_2_ production in *Monilinia fructicola* (Verde-Yáñez et al. 2023). Intracellular Ca^2+^ in the spores of *Onoclea sensibilis* increased from 0.1 to 10 μmol in response to red light exposure at 2.4 J m^−2^ s^−1^ (Wayne and Hepler 1985). In addition, a transient increase of intracellular ATP content was observed in *Trichoderma viride* upon white light exposure (Farkavš et al. 1985). Recently, nitric oxide (NO) has emerged as a new signaling molecule that modulates fungal growth, development and the biosynthesis of fungal secondary metabolites (Zhao et al. 2020). NO generation was also observed in fungi during light exposure, such as in *Trichophyton rubrum*, where intense pulsed light at 420 nm upregulated nitric oxide synthase (NOS) to synthesize NO, leading to fungal growth inhibition (Huang et al. 2019). Experiments using nitric oxide donor (sodium nitroprusside, SNP or S-nitrosoglutathione, GSNO) and NOS inhibitor (L-nitroarginine) demonstrated that NO inhibited the light-stimulated formation of conidia of *Neurospora crassa* (Ninnemann and Maier 1996; Filippovich et al. 2019). However, there have been no reports regarding NO generation in fungi induced by red light or its signaling roles in fungal secondary metabolite biosynthesis. Therefore, as a follow-up to our efforts to promote *Shiraia* HA production by light (Ma et al. 2019a) and understand the physiological roles of NO during the abiotic elicitation on *Shiraia* fungi (Li et al. 2020; Ma et al. 2021), we therefore wish to investigate red light-induced NO generation and its relationship with other eliciting responses, including ROS production, Ca^2+^ fluxes, extracellular ATP (eATP) levels, and *Shiraia* PQ biosynthesis. Additionally, a novel strategy involving combined elicitation with SNP (a NO donor) and red light is established for biotechnological production of hypocrellins in mycelium cultures.

## Material and methods

### Strains and culture conditions

The fungal strain *Shiraia* sp. S9 was isolated in our Lab (Ma et al. 2019b) and registered in China General Microbiological Culture Collection Center with accession number CGMCC16369. The fungal culture was maintained on potato dextrose agar (PDA; 200 g/L potato, 20 g/L glucose, 15 g/L agar) slants at 4 °C. For inoculation, spore suspension (4 mL of 10^7^ spores/mL) of *Shiraia* sp. S9 from PDA slants was transferred to 150 mL Erlenmeyer flasks containing 50 mL of liquid medium (potato, 100 g/L; starch, 20 g/L; NaNO_3_, 4 g/L; KH_2_PO_4_, 1.5 g/L; CaCO_3_, 0.5 g/L; VB1, 0.01 g/L; pH 6.3) for seed culture and incubated at 28 °C with shaking at 200 rpm for 2 days. The seed culture (10%, v/v) was poured into a 150 mL Erlenmeyer flask containing 50 mL of the same liquid medium for production culture (Sun et al. 2017).

### Red light treatment

For red light exposure, *Shiraia* cultures were exposed to red light (627 nm, 200 lx) for 8 days at 28 °C in an illumination incubator (ZD-8802, Hualida, Suzhou, China). The LED lamps (XYC-T5001, Xiaoyecao Photoelectric Technology Co., Ltd., Shenzhen, China) with a wavelength of 627 nm were installed on the incubator, and the light intensity was adjusted to 200 lx. More details of light conditions refer to our previous study (Ma et al. 2019a). For the control (dark treatment), flasks were wrapped with aluminum foil. For SNP treatment, SNP was dissolved in sterilized distilled water to make a 100 mM stock solution and filter sterilized. The stock solution was then added to the liquid medium to a final concentration of 5 μM. For the combined treatment, mycelia were exposed to red light and treated with SNP (1–20 μM) on different day (day 1–5) during 8 day cultures. The experiments were carried out in shake-flask cultures using 150 mL Erlenmeyer flasks containing 50 mL medium on a rotary shaker at 200 rpm and at 28 °C. The treatments consisted of triplicate independent repeats (ten flasks per replicate) and all results were expressed as mean ± standard deviation (SD).

### Observation of fungal morphology and conidia quantification

The fungal morphology of S9 was observed by a stereoscopic microscope (SMZ1000, Nikon, Tokyo, Japan), and the pellet diameter was measured in triplicates at different cultivation times (days 1–8). For spores count, the spore suspension was prepared by washing 8-day-old S9 with sterile water and the conidia number was determined using a hemocytometer under a microscope (CX21, Olympus, Tokyo, Japan). To observe the mycelial branches, a sterile coverslip was inserted on the periphery of a 3-day-old-S9 strain and continued to cultivate for 2 days at 28 ℃. The coverslip was removed and the hypha branching was observed under an inverted fluorescence microscope (TS2R-FL, Nikon, Tokyo, Japan). The determination of distance between two branches refers to the research by Ziv et al. (2013).

### Determination of NO levels and NOS, nitrate reductase (NR) activity

NO production was determined using the NO-specific fluorescent probe 4,5-diaminofluorescein diacetate (DAF-2 DA, Sigma-Aldrich, St. Louis, MO, USA) (Turrion‐Gomez and Benito 2011). Mycelia were harvested and washed 3 times with sterilized distilled water. After washing, mycelia were incubated in darkness with 10 μM DAF-2 DA for 30 min at 28 °C. The fluorescence intensity was measured using a fluorescence microscope (CKX41, Olympus, Tokyo, Japan) with an excitation/emission wavelength of 470/525 nm. NO levels in hyphae were determined using a Nitric Oxide Assay Kit (Beyotime Biotechnology, Nanjing, China) (Li et al. 2020). The activity of NOS and NR was measured following the manufacturer’s instructions of the NOS Kit and the NR Kit (Nanjing Jiancheng Institute of Bioengineering, Nanjing, China). The protein concentration was determined using the Enhanced BCA Protein Assay Kit (Beyotime Biotechnology, Shanghai, China). Both NO contents and the activity of NOS and NR were measured in the shake-flask cultures. The treatments consisted of triplicate independent repeats (ten flasks per replicate).

### cGMP determination

cGMP content in mycelia was determined using an ELISA Kit (Jiangsu Meimian Industrial Co., Ltd., Yancheng, China). Fresh mycelia were ground on the ice at a ratio of tissue (g): 0.01 M PBS (pH 7.2–7.4) (mL) of 1: 9, and tissue homogenate was centrifuged at 3000 rpm/min for 20 min at 4 ℃. The supernatant was used for cGMP content and protein concentration determination. The NO scavenger 2-(4-carboxyphenyl)-4,4,5,5-tetramethylimidazoline-1-oxyl-3-oxide (cPTIO) at 100 μM and soluble guanylate cyclase (sGC) inhibitor NS-2028 at 20 μM were added 30 min prior to the red light treatment.

### Detection of Ca^2+^, H_2_O_2_ and extracellular ATP

Intracellular Ca^2+^ levels were measured using the Fluo-3-AM (Beyotime Biotech., Haimen, Jiangsu, China) as a probe. The fungal pellets cultured under different conditions were incubated with 5 µM Fluo-3-AM for 2 h. The mycelia were washed 3 times with PBS and were photographed under fluorescence microscopy (BX51, Olympus, Tokyo, Japan) with excitation at 480 nm and emission at 515 nm. The inhibitors of EGTA (Ca^2+^ chelator, 5 mM) and La^3+^ (membrane channel blocker, 2 mM) were added 30 min before the red light irradiation. The H_2_O_2_ content was determined as Mirshekari et al. (2019) described with some modifications. Briefly, fungal mycelia (300 mg) were ground into homogenates with 4 mL of 0.1% trichloroacetic acid in ice bath and then centrifuged at 12000 rpm for 20 min at 4 °C. The supernatant (0.5 mL) was diluted with 0.5 mL of potassium phosphate buffers (10 mM, pH 7.0) and 1 mL of potassium iodide (10 M). Finally, the absorbance of the mixture was measured at 390 nm by a Shimadzu UV-2600 spectrophotometer (Kyoto, Japan). For eATP detection, the fermentation broth cultivated for different times was collected and measured using a luciferin-luciferase ATP assay kit (Beyotime Biotech., Haimen, Jiangsu, China) according to Wu et al. (2008). The eATP antagonists purinoceptor inhibitor pyridoxalphosphate-6-azophenyl-2′, 4′-disulfonic acid (PPADS, Abcam, Cambridge, MA, USA) at 10 µM and a specific inhibitor of membrane purinoceptors inhibitor reactive blue (RB, Yuanye Biotech., Shanghai, China) at 10 µM were added 30 min prior to the red light treatment.

### Membrane permeabilization assay

The membrane permeability was measured using a high-affinity nucleic acid stain fluorescent dye SYTOX Green (Molecular Probes, Eugene, Oregon, USA). The mycelia cultured for 5 days were treated with 0.5 μM SYTOX Green for 30 min and photography through a fluorescent microscope (CKX41, Olympus, Japan) with an excitation/emission wavelength of 488/538 nm. The NO donor SNP (5 μM) and scavenger cPTIO (100 μM), and sGC inhibitor NS-2028 (20 μM) were added 30 min prior to the red light treatment.

### PQ extraction and quantification

The fungal PQ extraction in the mycelium culture refers to our previous study (Lei et al. 2017). Therein, the PQ contents were detected by the reverse-phase Agilent 1260 HPLC system (Agilent, Wilmington, NC, USA) with Agilent HC-C18 column (250 × 4.6 mm) (Agilent, Santa Clara, CA, USA) with a mobile phase (acetonitrile: water at 65: 35, v/v). The injection volume was 10 μL with the flow rate 1 ml/min, and the UV detection wavelength was 465 nm (Tong et al. 2017). HPLC chromatograms of PQ standards were presented in Additional file 1: Fig. S1. Total hypocrellin A production refers to the sum of intracellular and extracellular hypocrellin A.

### Quantitative real-time PCR analysis

The primer of genes related to the sporulation (Ma et al. 2019a; Zhao et al. 2021b), genes for HA biosynthesis and internal reference gene (18S ribosomal RNA) are listed in Additional file 1: Table S1. The real-time quantitative PCR (RT-qPCR) was measured in the CFX96-C1000 Touch Real-Time PCR Detection System (Bio-Rad, Hercules, CA, USA) for gene transcription analysis.

### Statistical analysis

All experimental data were performed in triplicate independent experiments. Data were analyzed using one-way analysis of variance (ANOVA) with Dunnett′s multiple-comparison tests and student’s *t*-test. All the experimental results are expressed as mean ± standard deviation (SD) of triplicate experiments. Statistical significance was defined as *p* < 0.05.

## Results

### Effect of red light on fungal growth and PQ production

**Fig. 1:**
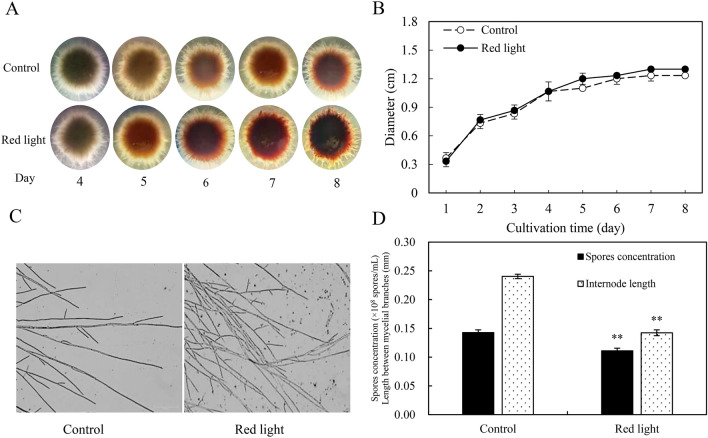
Effects of red light on the growth and development of *Shiraia* sp. S9. **A** The pellet morphology (15 ×) and pellet diameters (**B**) during the culture. The liquid culture of S9 was incubated in the dark or red light treatment at 28 ℃ and 150 r/min. The intensity of red light (627 nm) was 200 lx. **C** The mycelial morphology (400 ×) under the dark or red light treatment. **D** The length between mycelial branches and spore concentration of S9. Values are mean ± SD from three independent experiments (^**^p < 0.01 vs. dark control, ten flasks or plates per replicate)

To explore the influence of red light on the production of PQs, *Shiraia* sp. S9 was inoculated on the PDA plate or in mycelium culture under red light treatment. The intensity (200 lx) and exposure time (24 h/day) for red light were chosen based on our previous study (Ma et al. 2019a). After red light treatment, the fungal pellets became darker and tighter in the liquid cultures (Fig. 1A), but there was no significant difference in the pellet diameter (Fig. 1B). Under red light exposure, the number of mycelial branches was increased and the branching distance was shortened by 41.7%, whereas the spore formation was inhibited and the conidia count decreased by 22.2% compared to the dark control group (Fig. 1C, D). In the liquid culture, both pH value and residual sugar content remained unaltered following red light treatment (Additional file 1: Fig. S2).

**Fig. 2:**
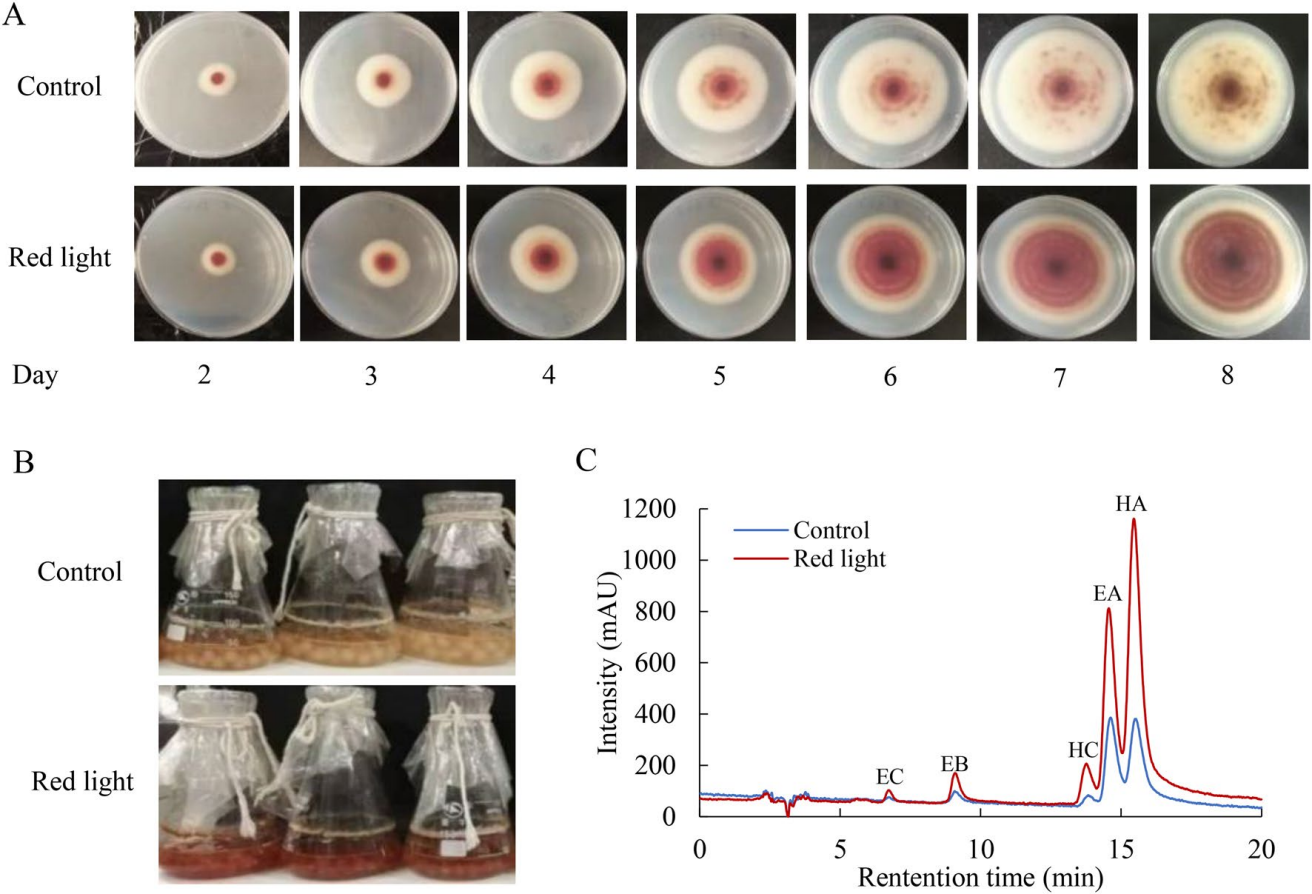
Effects of red light on morphology and perylenequinone (PQ) production of *Shiraia* sp. S9. **A** The solid-state culture of *Shiraia* sp. S9. The PDA plate was cultivated at 28 ℃ under dark (control group) or red light (treatment group) conditions, respectively. The intensity of red light (627 nm) was 200 lx. **B** The liquid culture of *Shiraia* sp. S9. The culture was maintained in a 150 mL flask containing 50 mL medium at 150 r/min and 28℃ and the photos were taken on day 3. **C** The chromatogram of individual PQs in the mycelium. Values are mean ± SD from three independent experiments (ten flasks or plates per replicate)

Although the application of red light did not elicit significant effects on fungal growth (pellet diameter) in liquid culture (Fig. 1B), the secretion of red pigments by the fungus was promoted in the plate (Fig. 2A). Furthermore, in liquid cultures, the exposure to red light notably boosted the production of red PQ pigments, both in the mycelium (Fig. 2B, C) and cultural broth (Table 1). Specifically, in mycelium culture, the content of individual PQs such as HA, HC, elsinochrome A (EA), EB and EC exhibited substantial increases, with fold changes of 3.1, 3.6, 2.0, 1.9 and 2.1, relative to the dark control (Table 1).

**Table 1:** Effects of red light on the individual perylenequinone (PQ) production in liquid culture of *Shiraia* sp. S9*

	HA	HC	EA	EB	EC
Intracellular PQs (mg/g DW)					
Control	9.64 ± 0.21	0.97 ± 0.26	1.71 ± 0.21	0.29 ± 0.025	0.14 ± 0.02
Red light	30.15 ± 0.72^**^	3.91 ± 0.04^**^	2.39 ± 0.25^*^	0.46 ± 0.05^**^	0.21 ± 0.02^*^
Red light + SNP	33.87 ± 1.97^#^	4.18 ± 0.16^#^	2.56 ± 0.31	0.50 ± 0.04	0.23 ± 0.01
Red light + cPTIO	27.30 ± 1.48^#^	3.53 ± 0.20^#^	1.78 ± 0.18^#^	0.98 ± 0.10^##^	0.42 ± 0.06^##^
Red light + NS-2028	15.07 ± 3.79^##^	2.23 ± 0.48^#^	1.68 ± 0.16^##^	0.21 ± 0.04	0.15 ± 0.02
Extracellular PQs (mg/L)					
Control	1.08 ± 0.06	0.36 ± 0.02	0.70 ± 0.08	0.21 ± 0.02	0.13 ± 0.02
Red light	2.56 ± 0.20^**^	0.92 ± 0.09^**^	2.40 ± 0.06^**^	0.49 ± 0.11^*^	0.36 ± 0.10^*^
Red light + SNP	3.86 ± 0.34^##^	1.46 ± 0.05^##^	1.79 ± 0.04^##^	0.35 ± 0.06	0.24 ± 0.02
Red light + cPTIO	2.03 ± 0.17^#^	0.66 ± 0.02^#^	1.50 ± 0.18^##^	0.27 ± 0.04^#^	0.16 ± 0.03^#^
Red light + NS-2028	1.97 ± 0.12^##^	0.56 ± 0.04^##^	1.38 ± 0.13^##^	0.27 ± 0.01^##^	0.17 ± 0.02^#^

^*^The SNP (5 μM), cPTIO (100 μM) and NS-2028 (20 μM) were added to the culture 30 min before the red light treatment. The intensity of red light (627 nm) was 200 lx. The culture was maintained in a 150 mL flask containing 50 mL medium at 150 r/min and 28 ℃ for 8 days. The treatments consisted of triplicate independent repeats (ten flasks per replicate, ^*^p < 0.05, ^**^p < 0.01 vs. control; ^#^*p* < 0.05, ^##^*p* < 0.01 vs. red light treatment)

### Red light-induced NO generation

**Fig. 3:**
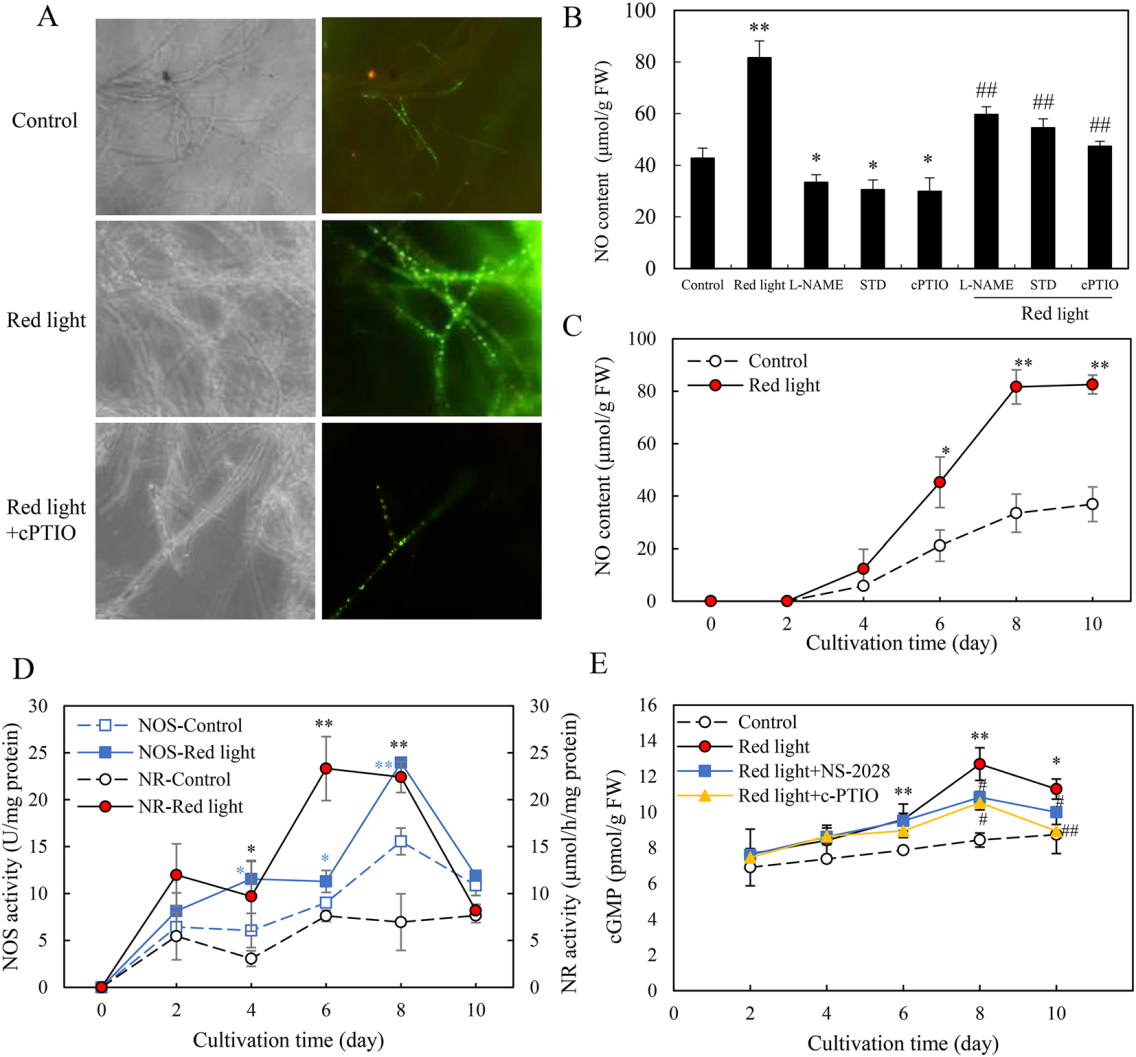
Effects of red light on NO generation of *Shiraia* sp. S9 in liquid culture. **A** Bright-field image (left) and fluorescence microscopy of DAF-2 DA-stained mycelia (right) (400 ×) in the cultures. cPTIO (100 μM) were added 30 min before red light treatment and the photo was taken on day 8. The intensity of red light (627 nm) was 200 lx. **B** The NO content in the mycelium. l-NAME (100 μM), STD (100 μM) and cPTIO (100 μM) were added 30 min before red light treatment respectively, and the mycelia were harvested on day 8. **C** Time course of NO content. **D** NOS and NR activity in *Shiraia* sp. S9. **E** cGMP content in *Shiraia* sp. S9. NS-2028 (20 μM) and cPTIO (100 μM) were added 30 min prior to the red light treatment. The culture was maintained in a 150 mL flask containing 50 mL medium at 28 ℃ and 150 r/min. Values are mean ± SD from three independent experiments. (^*^p < 0.05 and ^**^p < 0.01 vs. control. ^##^*p* < 0.01 vs. red light treatment, ten flasks per replicate)

In our investigation of the impact of red light on NO generation in *Shiraia* sp. S9, we used the NO-specific fluorescent probe DAF-2 DA as previously described (Turrion‐Gomez and Benito 2011). As shown in Fig. 3A, a conspicuous green fluorescence of DAF-2 DA appeared in the mycelia after red light treatment. The NO content increased to 1.9 times compared to the dark control (Fig. 3B). Notably, when the culture medium was pretreated with NO scavenger cPTIO, red light-induced fluorescence markedly diminished (Fig. 3A). Furthermore, the addition of NOS inhibitor *N*ω-nitro-l-arginine methyl ester (l-NAME) and NR inhibitor sodium tungstate dehydrate (STD) resulted in a suppression of NO content in mycelia by 27.0% and 33.3%, respectively (Fig. 3B). The time course of NO generation revealed that NO level began to elevate on day 4 after red light treatment and reached the highest point at 81.7 μmol/g FW on day 8, about 2.4-fold higher than the control (Fig. 3C). To further investigate on the sources of NO production, we assessed the enzyme activity of NOS and NR. Under red light treatment, both NOS and NR activity had a significant increase, reaching their peak value (24.0 U/mg protein of NOS and 23.3 U/mg protein of NR) on day 8 and day 6, respectively (Fig. 3D). Chen et al. (2022) reported that NO was involved in the l-arginine-induced PQ formation through the sGC pathway in *Shiraia* sp. Slf14(w). However, the corresponding gene encoding sGC was not found in *Shiraia* genome. In an effort to investigate on the involvement of sGC-cGMP pathway during red light treatment, we used sGC inhibitor NS-2028 and measured the content of cGMP. Red light treatment induced a 50.5% increase in cGMP content, reaching 12.7 pmol/g fresh weight (FW) on day 8. However, this increase was mitigated in the presence of the sGC inhibitor NS-2028 or the NO scavenger cPTIO (Fig. 3E). These findings collectively suggest that NO-sGC-cGMP pathway could be involved in the elicitation of red light.

### NO involved in the effects of red light on *Shiraia* growth

**Fig. 4:**
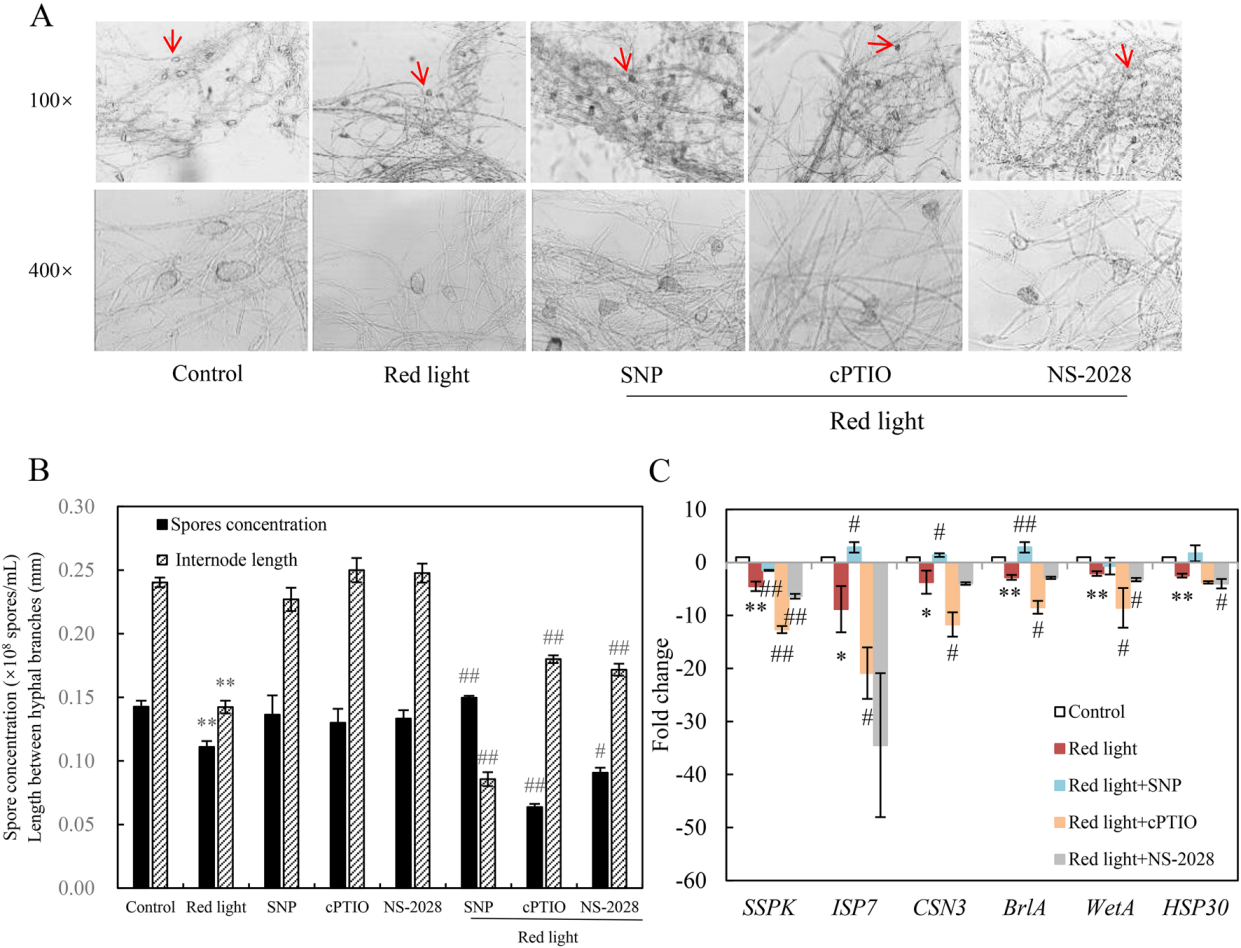
Effects of NO on the growth and development of *Shiraia* sp. S9 under the red light. **A** The morphologic characteristics of S9. The red arrow indicates pycnidium. SNP (5 μM), cPTIO (100 μM) and NS-2028 (20 μM) mixed with PDA medium were poured into a Petri dish and the plate was incubated at 28 ℃ for 8 days. The intensity of red light (627 nm) was 200 lx. **B** The number of spores and the length between mycelial branches of S9. **C** The relative expression levels of genes associated with sporulation. The culture was maintained in a 150 mL flask containing 50 mL medium at 28 ℃ and 150 r/min under the dark or red light treatment. Values are mean ± SD from three independent experiments (^*^p < 0.05 and ^**^p < 0.01 vs. control. ^#^*p* < 0.05 vs. red light treatment, ten plates per replicate)

Red light treatment reduced both the pycnidia production and spore numbers, which were subsequently restored by NO donor SNP (Fig. 4A). The NO scavenger cPTIO or sGC inhibitor NS-2028 further inhibited the spore concentration (Fig. 4B). Additionally, the length between mycelial branches was shortened by red light and the addition of SNP further decreased the branching length. Conversely, the addition of cPTIO or NS-2028 led to an increase of branching length when compared to red light treatment alone (red light + cPTIO or NS-2028 vs*.* red light in Fig. 4B). Furthermore, based on our previous transcriptional analysis of *S. bambusicola* S8 under red light (Ma et al. 2019a), we analyzed the expression levels of genes related to sporulation, including sexual differentiation process protein (*ISP7*), cleistothecium development (*CSN3*), stage v sporulation protein k (*SSPK*), 30 kDa heat shock protein (*HSP30*), and transcriptional factor *BrlA* and *WetA*. These genes have been reported to be responsive to external NO application and be also related to fungal conidiation (Boylan et al. 1987; Zhao et al. 2021b). In our study, their expressions were significantly down-regulated by red light, about 4.5-, 8.8-, 3.7-, 2.8-, 2.1-, 2.5-fold relative to the control, respectively. Importantly, the suppressed expressions of these genes were partially restored after the addition of SNP, but were further suppressed by cPTIO and NS-2028 (Fig. 4C). These results indicated the involvement of NO and the sGC-cGMP pathway in the regulation of fungal growth and development of *Shiraia* sp. S9 under red light conditions.

### NO involved in red light-induced fungal membrane permeability

**Fig. 5:**
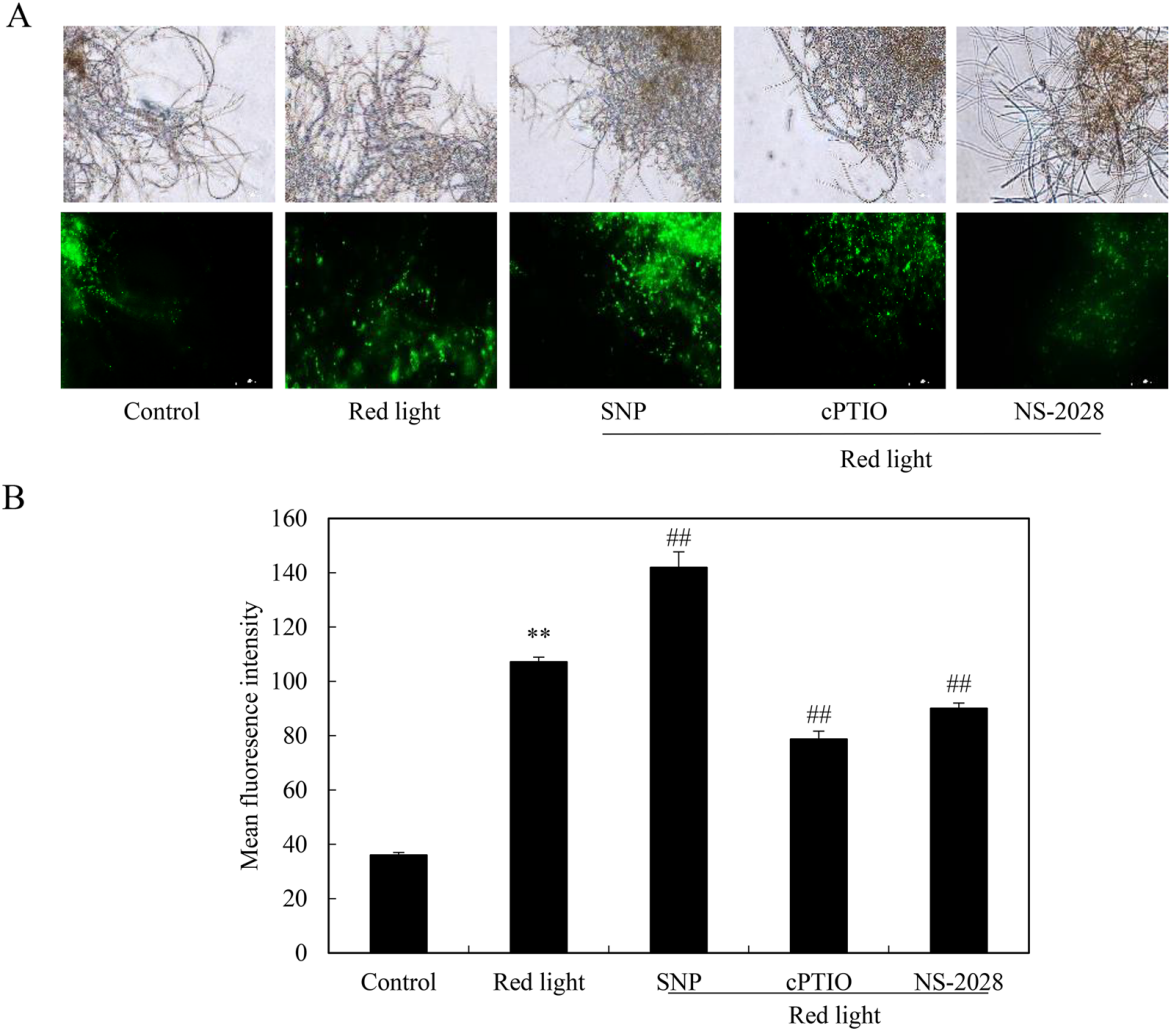
Effects of NO on hyphal cell membrane permeability of *Shiraia* sp. S9 under the red light. **A** The integrity of mycelial cell membrane. The mycelia cultured for 5 days were treated with 0.5 μM SYTOX Green for 30 min and photography through a fluorescent microscope. SNP (5 μM), cPTIO (100 μM) and NS-2028 (20 μM) were added 30 min prior to the red light treatment, respectively. The intensity of red light (627 nm) was 200 lx. **B** The mean fluorescence intensity. The culture was maintained in a 150 mL flask containing 50 mL medium at 28 ℃ and 150 r/min under the dark or red light treatment. Values are mean ± SD from three independent experiments (*p < 0.05 and **p < 0.01 vs. control. ^#^*p* < 0.05 vs. red light treatment, ten flasks per replicate)

To examine the impact of red light on the membrane permeability of *Shiraia* sp. S9, we employed SYTOX Green, a high-affinity nucleic acid stain fluorescent dye, as described by Thevissen et al. (1999). Following 5 days of exposure to red light, a pronounced green fluorescence signal was evident (Fig. 5A), indicating an enhanced membrane permeability. Remarkably, the red light-induced fluorescent signal was further intensified upon the addition of the NO donor SNP, about 1.3-fold compared to the group subjected to red light alone (red light + SNP vs. red light in Fig. 5). Conversely, the addition of NO scavenger cPTIO or sGC inhibitor NS-2028 attenuated red light-induced fluorescence (Fig. 5A, B), thereby suggesting the involvement of red-light induced NO signal in the increased membrane permeability of *Shiraia* sp. S9.

### Red light-induced ROS, Ca^2+^ and eATP signals

**Fig. 6:**
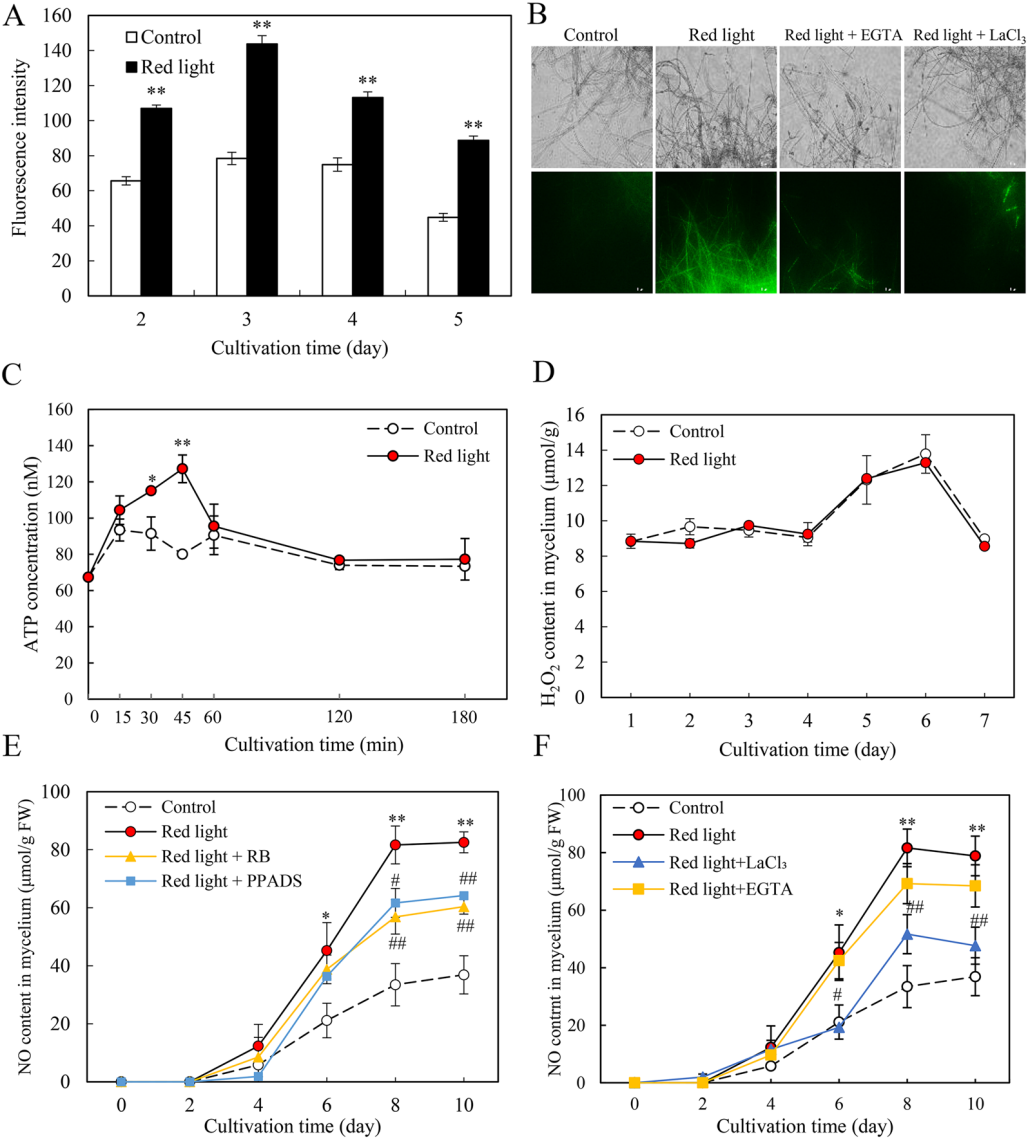
Effects of red light on the signal molecules in *Shiraia* sp. S9. **A** The relative fluorescence intensity of Ca^2+^ in S9. **B** Changes in the fluorescence of Ca^2+^. bright-field image (above) and fluorescence microscopy (below) of Fluo-3 AM-stained mycelia (400 ×). EGTA (5 mM) and LaCl_3_ (2 mM) were added 30 min prior to the red light, respectively. The intensity of red light (627 nm) was 200 lx. The red light-induced eATP release (**C**) and H_2_O_2_ content (**D**) in the mycelium of S9. **E** The effect of eATP signal on NO content. PPADS (10 μM), RB (10 μM) were added 30 min prior to the red light treatment, respectively. **F** The effect of Ca^2+^ signal on NO content. Values are mean ± SD from three independent experiments (*p < 0.05 and **p < 0.01 vs. control. ^#^*p* < 0.05 and ^##^*p* < 0.01 vs. red light treatment, ten flasks per replicate)

We conducted assessments of the other signaling molecules, including ROS, Ca^2+^ and extracellular ATP (eATP) as part of the early signaling events in *Shiraia* cultures during elicitation (Lu et al. 2019; Li et al. 2021). Notably, an increase in Ca^2+^ content induced by red light was observed, as evidenced by the intense green fluorescence exhibited by Fluo-3 AM (Fig. 6A). The increased Ca^2+^ content was further substantiated through the reduction of the fluorescence intensity upon the application of the Ca^2+^ chelator EGTA and membrane channel blocker La^3+^ (Fig. 6B). Furthermore, red light induced eATP content to 127 nM after 45 min, about 1.6-fold increase compared to the control (Fig. 6C). However, there was no significant change in H_2_O_2_ contents during the red light treatment (Fig. 6D). To delve into the interaction between Ca^2+^ or eATP signals and NO, we employed eATP antagonists (the purinoceptor inhibitor PPADS and RB) and Ca^2+^ antagonists (EGTA and LaCl_3_), respectively. The red light-induced NO production was markedly inhibited by eATP antagonists (PPADS or RB) (Fig. 6E). Moreover, pretreatment of EGTA or LaCl_3_ resulted in a 15.2% and 36.7% decrease in NO content, respectively (EGTA or LaCl_3_ + Red light vs. Red light in Fig. 6F). These findings collectively indicate the involvement of both eATP and Ca^2+^ in the regulation of NO production during red light treatment.

### The mediation of NO in red light-induced *Shiraia* PQ biosynthesis

**Fig. 7:**
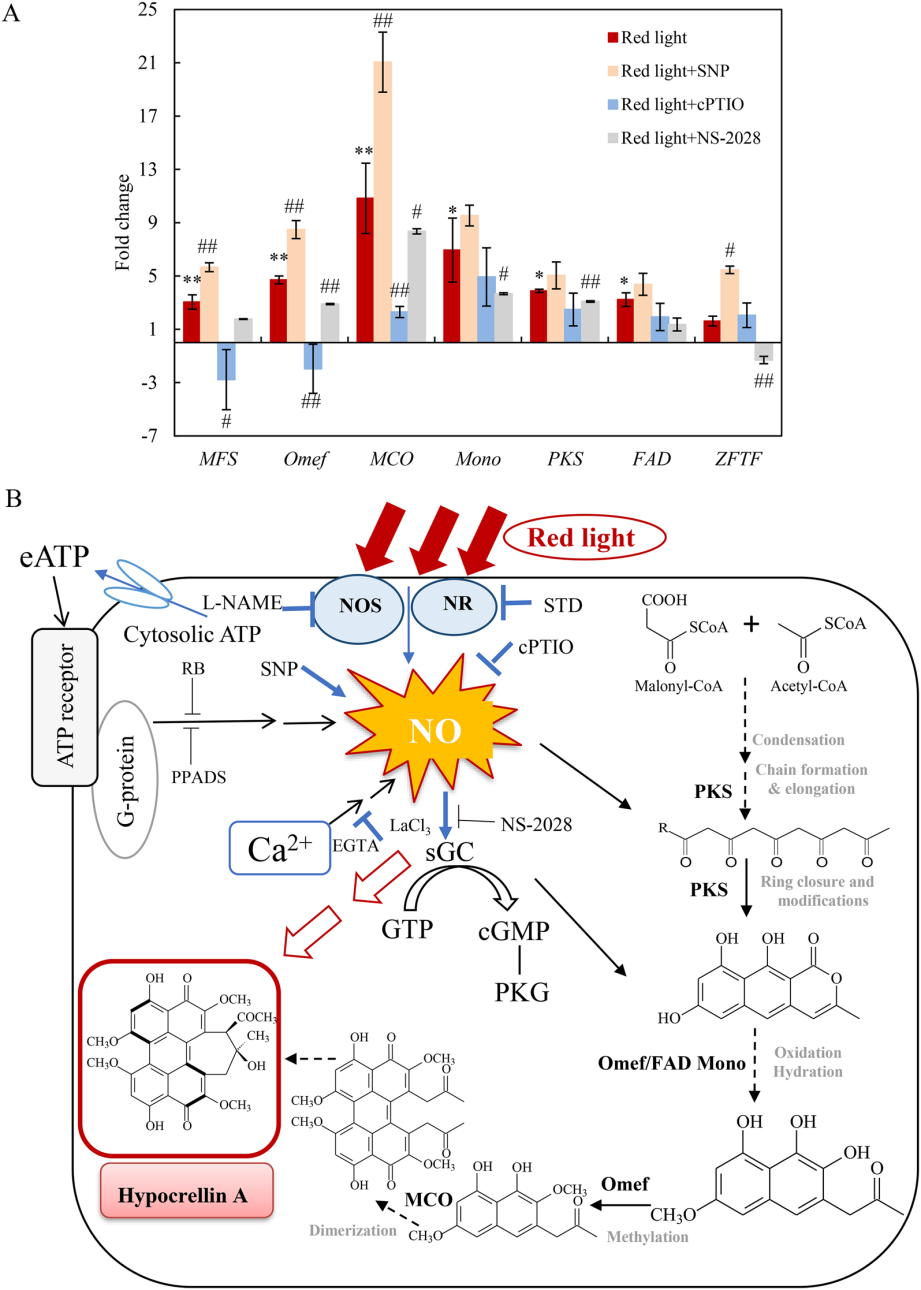
Effects of NO on the expression of perylenequinone (PQ) biosynthetic genes under red light treatment (**A**). SNP (5 μM), cPTIO (100 μM) and NS-2028 (20 μM) were added to the culture 30 min before red light treatment, respectively. The intensity of red light (627 nm) was 200 lx. Values are mean ± SD from three independent experiments. (^*^p < 0.05 and ^**^p < 0.01 vs. control. ^#^*p* < 0.05 and ^##^*p* < 0.01 vs. red light treatment, ten flasks per replicate). **B** A schematic diagram of red light-induced HA biosynthesis of *Shiraia* sp. S9. *NO* nitric oxide, *eATP* extracellular ATP, *EGTA* Ca^2+^ chelator, *LaCl*_*3*_ membrane channel blocker, *RB* an inhibitor of eATP signal transduction across the plasma membrane, *PPADS* the purinoceptor inhibitor, *sGC* soluble guanylate cyclase, *NS-2028* sGC inhibitor

Exposure to red light significantly enhanced the accumulation of PQs (Fig. 2, Table 1). In conjunction with this observation, we investigated the expression levels of genes associated with PQ synthesis, including major facilitator superfamily (*MFS*), *O*-methyl-transferase (*Omef*), multicopper oxidase (*MCO*), monooxygenase (*Mono*), polyketide synthase (*PKS*), FAD/FMN-containing dehydrogenase (*FAD*) and zinc finger transcription factor (*ZFTF*) (Zhao et al. 2016). When the mycelium culture was exposed to red light, the transcriptional levels of these genes were up-regulated, with fold increases of 3.0-, 4.7-, 10.8-, 6.9-, 3.9-, 3.2- and 1.6-fold, respectively (Fig. 7A). Furthermore, we delved into the role of NO in red light-induced PQ production of *Shiraia* sp. S9. The application of the NO donor SNP resulted in a further promotion of intracellular PQ contents, particularly HA (12.33% increase). However, both intracellular PQs (HA, HC and EA) and extracellular PQs were inhibited by the NO scavenger cPTIO or sGC inhibitor NS-2028 (Table 1). The transcription levels of genes related to PQ synthesis were similarly enhanced by SNP but repressed by cPTIO or NS-2028 (Fig. 7A). These findings collectively suggest that NO and its sGC-cGMP pathway play a pivotal role in the enhanced PQ production in *Shiraia* sp. S9 cultures exposed to red light.

### Combined elicitation of red light and SNP on *Shiraia* HA production

**Fig. 8:**
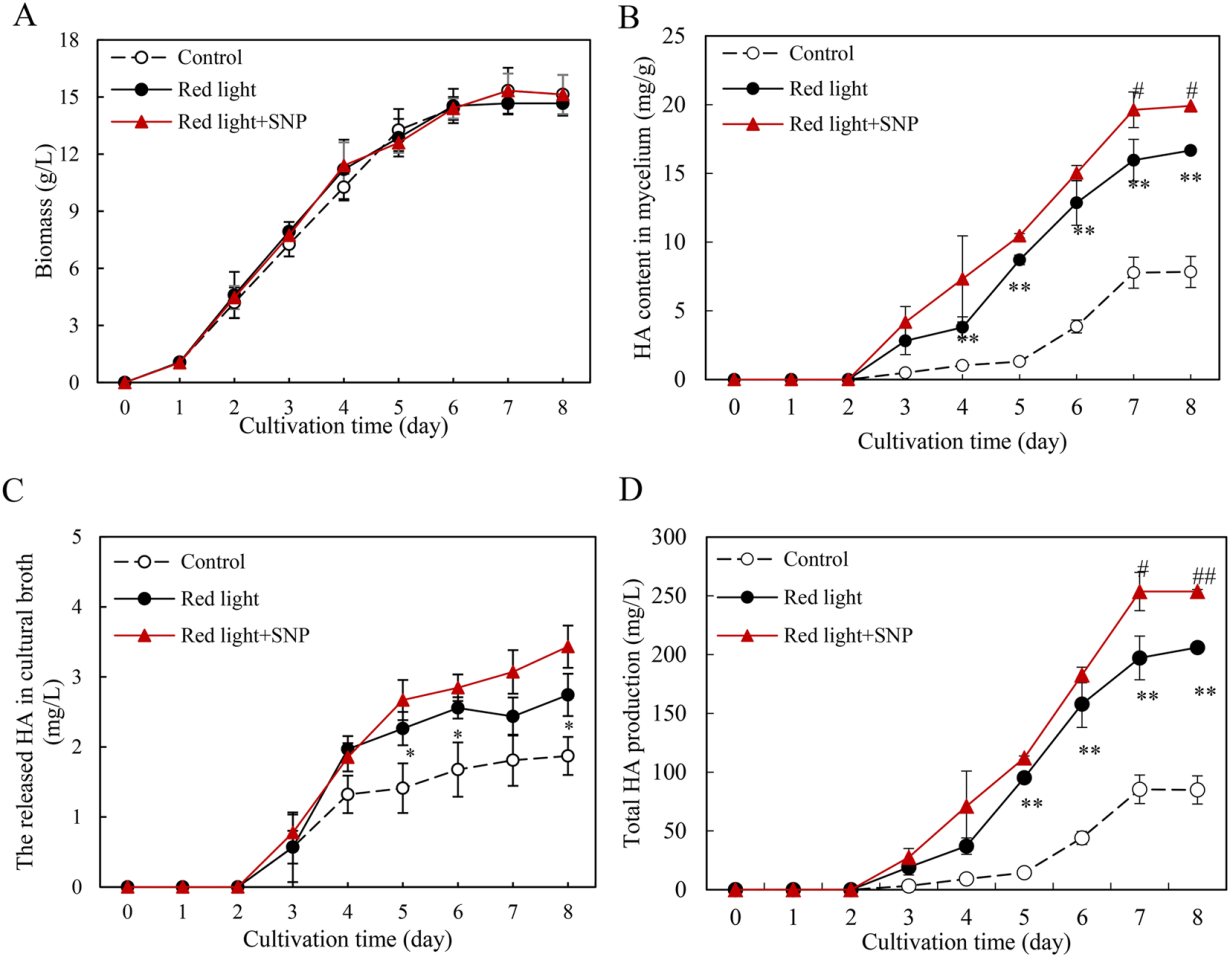
Time profiles of fungal biomass (**A**), HA content in mycelium (**B**), the released HA in cultural broth (**C**) and total HA production (**D**) of *Shiraia* sp. S9. The culture was maintained in a 150 mL flask containing 50 mL medium at 150 r/min and 28 ℃ under the dark or red light treatment for 8 days. The intensity of red light (627 nm) was 200 lx. SNP (5 μM) was added on day 1 of culture, 30 min before the red light treatment. Values are mean ± SD from three independent experiments (*p < 0.05 and **p < 0.01 vs. control. ^#^*p* < 0.05 and ^##^*p* < 0.01 vs. red light treatment, ten flasks per replicate)

To enhance the production of HA, a key bioactive PQ in *Shiraia* mycelium cultures, we introduced SNP (5 μM) in the culture 30 min prior to initiating red light treatment according to our pre-experiment results (Additional file 1: Fig. S3, Fig. 4). Although the red light treatment or its combination with SNP (1, 5, 10 and 20 μM) did not yield significant changes in fungal biomass (Additional file 1: Fig. S3A), it did lead to a significant enhancement in HA production (Additional file 1: Fig. S3B–D). Furthermore, when subjected to the combined treatment of red light and 5 μM SNP on day 1, HA content in both the mycelium and cultural broth was stimulated significantly. Consequently, the total HA production was boosted by 38.6% compared to the red light treatment alone (Additional file 1: Fig. S3D). Subsequently, we introduced 5 μM SNP into the red light cultures at different time points (day 1–5). The choice of day 1 for SNP addition was based on its eliciting effects on HA production (Additional file 1: Fig. S4). Under the optimized elicitation conditions, where SNP (5 μM) was added on day 1 of the mycelium culture under red light exposure (200 lx), the total HA production reached 254 mg/L, about 3.0-fold increase over the dark control (Fig. 8).

## Discussion

*Shiraia* spp. are pathogenic fungi known to colonize bamboo branches in Eastern Asia (Morakotkarn et al. 2007). The *Shiraia* infection usually occurs in the brim of bamboo forest and its fruitbodies were formed at the shoot apex of bamboos (Li et al. 2009; Liu et al. 2012), implying a possible dependence on light for the fungal growth and development. Given that photoactive PQs play a significant role in fungal virulence and pathogenicity by generating ROS (Daub et al. 2013), it is reasonable to hypothesize that the PQ-producing fungi like *Shiraia, Cercospora* and *Elsinoё* species may exhibit sensitivity to light during growth and infections. Fungi typically generate ROS as a response to stress conditions induced by ultraviolet radiation (UV) and blue light exposure (Kim et al. 2013; Schumacher and Gorbushina 2020). In our previous study, it was observed that a light/dark shift (24: 24 h) or the intermittent blue light (6 h per day) at 200 lx stimulated HA production in *Shiraia* mycelium cultures, with concomitant induction of fungal ROS generation (Sun et al. 2018; Li et al. 2022). In the present study, red light (627 nm) at 200 lx was found to promote the accumulation of fungal PQs, including HA, HC and EA-EC in *Shiraia* sp. S9 (Table 1), however, there was no significant changes in H_2_O_2_ production in *Shiraia* mycelia under red light treatment (Fig. 6D). To the best of our knowledge, the only published work on fungal H_2_O_2_ production induced by red light (light-emitting diode, Philips 20W/T9/BLB) at 660 nm was conducted in *Monilinia fructicola* by Verde-Yáñez et al. (2023). In addition to ROS, NO is another signaling molecule in fungal responses to light exposure (Cánovas et al. 2016). NO has been shown to participate in the light-dependent regulation of conidiation in *Neurospora crassa* (Ninnemann and Maier 1996) and *Aspergillus nidulans* (Marcosa et al. 2020). Huang et al. (2017; 2019) reported that intense pulsed light (IPL) could induce NO generation in the dermatophyte *Trichophyton rubrum*, inhibiting its growth through nitrosative damage with ROS. Our current study demonstrates red light-induced NO generation (Fig. 3), and its regulatory effects on *Shiraia* growth, development (Fig. 4) and PQ production (Fig. 2, Table 1). Similar reports on induced NO generation have been found in *Shiraia* species treated with both biotic elicitors from *Aspergillum niger* (Du et al. 2015) and *Phytophthora boehmeriae* (Du et al. 2019), as well as abiotic elicitation such as Triton X-100 treatment (Li et al. 2020) or heat stress (Xu et al. 2023). Thus, NO may be one of important signals in the elicitation of *Shiraia* fungi by red-light exposure. Unlike the aforementioned elicitation methods, red light induced NO production in a longer duration (approximately 4–8 days) (Fig. 3C), and this elicitation by red light appeared to be independent of ROS in *Shiraia* cultures (Fig. 6D). This discrepancy likely arises from the different physiological responses to red light compared to other elicitors. NO can be synthesized through oxidative pathway mediated by NOS converting L-arginine to NO (Gorren and Mayer 2007). While the process of NO biosynthesis in fungi is not yet fully understood (Cánovas et al. 2016), l-arginine has been implicated in NO synthesis (Chen et al. 2022), and a NOS-like (NOSL) gene has been cloned from *Shiraia* sp. Slf14(w) (Xu et al. 2023). In our study, red light-induced NO production was inhibited by the NOS inhibitor (l-NAME), aligning with the role of NOS in *Shiraia.* Furthermore, the induced NO production was significantly suppressed by NR inhibitor STD (Red light + STD vs. Red light in Fig. 3B), suggesting the possible occurrence of a NR-dependent side reaction contributing to induced NO production in an alternative pathway (Yamasaki and Sakihama 2000). To our knowledge, this study represents the first report of fungal NO generation induced by red light and its effects on fungal growth and secondary metabolite biosynthesis.

After red-light exposure the fungal pellets of *Shiraia* sp. S9 had a dark tight core within the filamentous aggregation (Fig. 1A), suggesting alternations of fungal growth and PQ production. Although *Shiraia* biomass remained unchanged (Additional file 1: Figs. S3A, S4A), red light had inhibitory effects on fungal conidiation (Fig. 1D) and led to a reduction in the distance between hyphal branches (Fig. 1C, D), indicating that red light could be unfavorable for fungal growth and development. Röhrig et al. (2013) found that the spore germination of *A. nidulans* was significantly inhibited in the presence of red light, which was depended on the phytochrome FphA. The phytochrome FphA, light sensor VeA, CryA and the white collar (WC) complex (LreA, LreB, WC-1, 2) could mediate the transition of fungal asexual and sexual development under red light (Purschwitz et al. 2008). Therefore, sensing red light is complex signal-transduction processes for fungal development and metabolite biosynthesis. NO has been reported to play a role in the initiation and development of the spore production of *Coniothyrium minitans* (Gong et al. 2007). Zhao et al. (2021b) also observed up-regulation of conidiation-specific genes in response to SNP (0.2 mmol/L) and their suppression by the NO scavenger cPTIO. In our study, the induced NO could mitigate the inhibitory effects of red light on fungal growth (Fig. 4). NO can activate sGC, leading to the conversion of GTP to cGMP, which subsequently binds to protein kinase G (PKG) and initiates NO-cGMP-PKG signaling pathway to elicit the specific cellular responses (Zhao et al. 2020). When the sGC inhibitor NS-2028 was introduced into *Shiraia* cultures under red light*,* fungal conidiation was further suppressed and the distance of hyphal branches increased (red light + NS-2028 vs. red light in Fig. 4B, C). Simultaneously, cGMP production was induced by red light during the cultures (Fig. 3E), suggesting a regulatory role of sGC in fungal sporulation and hyphal growth. Furthermore, exogenous NO supplied by SNP not only promoted fungal conidiation (Fig. 4B), but also potentiated the red light-induced perylenequinone accumulation of *Shiraia* sp. S9 (Table 1, Additional file 1: Fig. S3, Fig. 4). Mycelium cultures exposed to red-light in the presence of a sGC inhibitor NS-2028 or a NO scavenger cPTIO significantly decreased the contents of individual PQs (Table 1). Therefore, our study suggests that NO can also mediate red light-induced PQ biosynthesis through the NO-sGC-GMP signaling pathway.

In addition to NO generation, red light exposure also induced the production of other signal molecules, such as eATP and Ca^2+^, during the early stage of mycelium culture (Fig. 6). Our study demonstrated an effective inhibition of fungal NO production by purinoceptor inhibitors (RB, PPADS) (Fig. 6E), as well as by the calcium chelator EGTA and the calcium channel blocker La^3+^ (Fig. 6F). These findings strongly suggest that eATP and Ca^2+^ play pivotal roles as key signals in mediating the physiological responses induced by NO in *Shiraia* sp. S9 under red light exposure. Our previous study showed that eATP acted as the intercellular signal to induce HA biosynthesis during co-culture of *Shiraia* sp. S9 with a bacterium *Pseudomonas fulva* SB1 (Li et al. 2021). The involvement of Ca^2+^/calmodulin (CaM) signaling in PQ production of *Shiraia* sp. Slf14 was also suggested, as evidenced by the effects of Ca^2+^ supplement and the use of Ca^2+^ sensor inhibitors (chlorpromazine and tacrolimus) (Liu et al. 2018). In the current study, we observed significant upregulation in the expressions of seven genes (*MFS*, *Omef*, *MCO*, *Mono*, *PKS*, *FAD* and *ZFTF*) from the hypocrellin synthesis gene cluster in response to red light (Fig. 7). Both the red light-induced expressions of HA biosynthetic genes (Fig. 7A) and HA production (Table 1) were markedly inhibited by the NO scavenger cPTIO. These results collectively suggest that NO can act as a signal molecule with eATP and Ca^2+^, working synergistically to enhance the biosynthesis of HA under red light conditions.

SNP, a NO donor has been utilized in plant cell cultures to stimulate production of plant secondary metabolites, such as hypericin, puerarin and catharanthine (Xu et al. 2005a, b; 2006). More recently, researchers have extended their efforts to use SNP as an elicitor in submerged mycelium cultures to enhance fungal metabolites, including the production of ganoderic triterpenoid in *Ganoderma lucidum* (Gu et al. 2017) and polyphenols in *Inonotus obliquus* (Zheng et al. 2009). In our previous work, we observed that the intracellular HA content of *Shiraia* sp. S9 increased by 73.31–178.96% when treated with 0.02 mM SNP on day 3 of a 9-day-culture (Ma et al. 2021). Additionally, Zhao et al. (2021a) reported that exogenous addition of 0.01 mM SNP promoted PQ production (HA and elsinochrome A) in *S. bambusicola* S4201 by 156% compared to the control. In the current study, we combine SNP treatment with red light exposure in the mycelium culture of *Shiraia* sp. S9 (Fig. 8). When 5 μM SNP was added on day 1 of the culture under red light exposure at 200 lx, fungal HA production increased to 254 mg/L, about 3.0-fold over the dark control. This approach using red-light treatment offers biotechnological advantages, particularly in fungal HA production in large-scale bioreactors with light‐emitting diodes. Further increases in HA production can be expected through the optimization of culture conditions and the refinement of the combined eliciting strategies.

## Conclusions

In conclusion, this study represents the first assessment of the mediating role of NO in the regulation of *Shiraia* conidiation, hyphal growth, and PQ production in response to red light exposure. Our investigation clearly showed that red light could inhibit fungal conidiation, increase hyphal branching and stimulate PQ accumulation in *Shiraia* sp. S9. The NO donor SNP potentiated red light-induced responses in fungal conidiation and PQ biosynthesis. Moreover, endogenous NO generation in the hyphae was induced by red light via NOS- or NR-dependent routes. The impact of red light on fungal conidiation and PQ production is partially mitigated upon the introduction of NO scavenger cPTIO or sGC inhibitor NS-2028, suggesting the NO-cGMP-PKG signaling pathway in *Shiraia* sp. after red light exposure. It is interesting to note that NO collaboratively functions as a signaling molecule with eATP and Ca^2+^, working synergistically to enhance PQ biosynthesis under red light conditions. NO generation induced by red light was further implicated in enhancing membrane permeability and upregulating transcript levels of PQ biosynthetic genes in *Shiraia* sp. S9, which led to the increase of both intracellular and extracellular PQ production. Importantly, the synergistic combination of red light exposure and NO donor SNP treatment amplified the stimulation of HA production in *Shiraia* mycelium culture. Our study provided a new understanding of NO signaling in biosynthesis of fungal metabolites, and shed new light on the signaling events during red light exposure. The new strategy of combined elicitation could be harnessed in photo-bioreactors for the potential biotechnological production of fungal secondary metabolites with pharmaceutical and industrial applications.

### Supplementary Information

**Additional file 1: Table S1.** Primers and relevant information of reference and target genes. F: forward primer, R: reverse primer. **Fig. S1.** HPLC chromatograms of PQ standards. 1. elsinochrome C; 2. elsinochrome B; 3. hypocrellins C; 4. elsinochrome A; 5. hypocrellins A. **Fig. S2.** Effects of red light on the pH value (A) and residual sugar (B) in *Shiraia* sp. S9. The culture was maintained in a 150 mL flask containing 50 mL medium at 28℃ and 150 r/min under the dark or red light treatment. The intensity of red light (627 nm) was 200 lx. Values are mean ± SD from three independent experiments. **Fig. S3.** Effects of different concentrations of SNP under red light treatment on biomass (A), HA content in mycelium (B), the released HA in cultural broth (C) and total HA production (D) of *Shiraia* sp. S9. The culture was maintained in a 150 mL flask containing 50 mL medium at 150 r/min and 28℃ under the dark or red light treatment for 8 days. The intensity of red light (627 nm) was 200 lx. SNP (1, 5, 10 and 20 μM) were added 30 min prior to the red light treatment. Values are mean ± SD from three independent experiments (*p< 0.05 and **p < 0.01 vs. control. ^#^*p* < 0.05 and ^##^*p* < 0.01 vs. red light treatment). **Fig. S4.** Effects of addition time of SNP (5 μM) under red light treatment on HA production of *Shiraia* sp. S9. The fungal dry biomass (A), HA content in mycelium (B), the released HA in cultural broth (C) and total HA production (D) in liquid culture. The culture was maintained in a 150 mL flask containing 50 mL medium at 150 r/min and 28℃ under the dark or red light treatment for 8 days. The intensity of red light (627 nm) was 200 lx. SNP (5 μM) was added on day 1-5 of culture, 30 min prior to the red light treatment. Values are mean ± SD from three independent experiments (*p < 0.05 and **p < 0.01 vs. control. ^#^*p* < 0.05 and ^##^*p* < 0.01 vs. red light treatment).

## Data Availability

The datasets generated and analyzed during this study are included in the published article [and its Additional files].

## References

[CR1] Boylan MT, Mirabito PM, Willett CE, Zimmerman CR, Timberlake WE (1987). Isolation and physical characterization of three essential conidiation genes from *Aspergillus nidulans*. Mol Cell Biol.

[CR2] Cánovas D, Marcos JF, Marcos AT, Strauss J (2016). Nitric oxide in fungi: is there NO light at the end of the tunnel?. Curr Genet.

[CR3] Chen YN, Xu CL, Yang HL, Liu ZY, Zhang ZB, Yan RM, Zhu D (2022). L-Arginine enhanced perylenequinone production in the endophytic fungus * Shiraia * sp. Slf14(w) via NO signaling pathway. Appl Microbiol Biotechnol.

[CR4] Daub ME, Herrero S, Chung KR (2013). Reactive oxygen species in plant pathogenesis: the role of perylenequinone photosensitizers. Antioxid Redox Signal.

[CR5] Du W, Liang JD, Han YF, Yu JP, Liang ZQ (2015). Nitric oxide mediates hypocrellin accumulation induced by fungal elicitor in submerged cultures of *Shiraia bambusicola*. Biotechnol Lett.

[CR6] Du W, Sun CL, Wang BG, Wang YM, Dong B, Liu JH, Xia JB, Xie WJ, Wang J, Sun JK, Liu XH, Wang HG (2019). Response mechanism of hypocrellin colorants biosynthesis by *Shiraia bambusicola* to elicitor PB90. AMB Express.

[CR7] Fanelli F, Schmidt-Heydt M, Haidukowski M, Susca A, Geisen R, Logrieco A, Mulè G (2012). Influence of light on growth, conidiation and fumonisin production by *Fusarium verticillioides*. Fungal Biol.

[CR8] Farkavš V, Sulová Z, Lehotský J (1985). Effect of light on the concentration of adenine nucleotides in *Trichoderma viride*. Folia Microbiol.

[CR9] Filippovich SY, Onufriev MV, Bachurina GP, Kritsky MS (2019). The role of nitrogen oxide in photomorphogenesis in *Neurospora crassa*. Appl Biochem Microbiol.

[CR10] Gao RJ, Xu ZC, Deng HX, Guan ZB, Liao XR, Zhao Y, Zheng XH, Cai YJ (2018). Influences of light on growth, reproduction and hypocrellin production by *Shiraia* sp. SUPER-H168. Arch Microbiol.

[CR11] Gong XY, Fu YP, Jiang DH, Li GQ, Yi XH, Peng YL (2007). L-Arginine is essential for conidiation in the filamentous fungus *Coniothyrium minitans*. Fungal Genet Biol.

[CR12] Gorren ACF, Mayer B (2007). Nitric-oxide synthase: a cytochrome P450 family foster child. Biochim Biophys Acta-Gen Subj.

[CR13] Gu L, Zhong X, Lian DH, Zheng YM, Wang HZ, Liu X (2017). Triterpenoid biosynthesis and the transcriptional response elicited by nitric oxide in submerged fermenting *Ganoderma lucidum*. Process Biochem.

[CR14] Huang H, Lv WB, Chen Y, Zheng XF, Hu Y, Wang RH, Huang ML, Tang HF (2017). The role of NADPH oxidase in the inhibition of *Trichophyton rubrum* by 420-nm intense pulsed light. Front Microbiol.

[CR15] Huang H, Huang ML, Lv WY, Hu Y, Wang RH, Zheng XF, Ma YT, Chen CM, Tang HF (2019). Inhibition of *Trichophyton rubrum* by 420-nm intense pulsed light: *In vitro* activity and the role of nitric oxide in fungal death. Front Pharmacol.

[CR16] Khiralla A, Mohammed AO, Yagi S (2022). Fungal perylenequinones. Mycol Prog.

[CR17] Kim K, Kook HS, Jang YJ, Lee WH, Kamala-Kannan S, Chae JC, Lee KJ (2013). The effect of blue-light-emitting diodes on antioxidant properties and resistance to *Botrytis cinerea* in tomato. J Plant Pathol Microbiol.

[CR18] Lei XY, Zhang MY, Ma YJ, Wang JW (2017). Transcriptomic responses involved in enhanced production of hypocrellin A by addition of Triton X-100 in submerged cultures of *Shiraia bambusicola*. J Ind Microbiol Biotechnol.

[CR19] Li XM, Gao J, Yue YD, Hou CL (2009). Studies on systematics, biology and bioactive substance of *Shiraia bambusicola*. Forest Res.

[CR20] Li XP, Wang Y, Ma YJ, Wang JW, Zheng LP (2020). Nitric oxide and hydrogen peroxide signaling in extractive *Shiraia* fermentation by Triton X-100 for hypocrellin A production. Int J Mol Sci.

[CR21] Li XP, Zhou LL, Guo YH, Wang JW (2021). The signaling role of extracellular ATP in co-culture of *Shiraia* sp. S9 and *Pseudomonas fulva* SB1 for enhancing hypocrellin A production. Microb Cell Fact.

[CR22] Li XP, Ji HY, Wang WJ, Shen WH, Wang JW (2022). Effects of blue light on hypocrellin A production in *Shiraia* mycelium cultures. Photochem Photobiol.

[CR23] Liu YX, Liu ZY, Wongkaew S (2012). Developing characteristics and relationships of *Shiraia bambusicola* with Bamboo. Songklanakarin J Sci Technol.

[CR24] Liu B, Bao JY, Zhang ZB, Yan RM, Wang Y, Yang HL, Zhu D (2018). Enhanced production of perylenequinones in the endophytic fungus *Shiraia* sp. Slf14 by calcium/calmodulin signal transduction. Appl Microbiol Biotechnol.

[CR25] Lu CS, Ma YJ, Wang JW (2019). Lanthanum elicitation on hypocrellin A production in mycelium cultures of *Shiraia bambusicola* is mediated by ROS generation. J Rare Earths.

[CR26] Ma YJ, Sun CX, Wang JW (2019). Enhanced production of hypocrellin A in submerged cultures of *Shiraia bambusicola* by red light. Photochem Photobiol.

[CR27] Ma YJ, Zheng LP, Wang JW (2019). Bacteria associated with *Shiraia* fruiting bodies influence fungal production of hypocrellin A. Front Microbiol.

[CR28] Ma YJ, Li XP, Wang Y, Wang JW (2021). Nitric oxide donor sodium nitroprusside-induced transcriptional changes and hypocrellin biosynthesis of *Shiraia* sp. S9. Microb Cell Fact.

[CR29] Marcosa AT, Ramosa MS, Schinkob T, Straussb J, Cánovasa D (2020). Nitric oxide homeostasis is required for light-dependent regulation of conidiation in *Aspergillus*. Fungal Genet Biol.

[CR30] Miller GG, Brown K, Ballangrud AM, Barajas O, Xiao Z, Tulip J, Lown JW, Leithoff JM, Allalunis-Turner MJ, Mehta RD, Moore RB (1997). Preclinical assessment of hypocrellin B and hypocrellin B derivatives as sensitizers for photodynamic therapy of cancer: progress update. Photochem Photobiol.

[CR31] Mirshekari A, Madani B, Golding JB (2019). Aloe vera gel treatment delays postharvest browning of white button mushroom (*Agaricus bisporus*). J Food Meas Charact.

[CR32] Miyake T, Mori A, Kii T, Okuno T, Usui Y, Sato F, Sammoto H, Watanabe A, Kariyama M (2005). Light effects on cell development and secondary metabolism in *Monascus*. J Ind Microbiol Biotechnol.

[CR33] Morakotkarn D, Kawasaki H, Seki T (2007). Molecular diversity of bamboo-associated fungi isolated from Japan. FEMS Microbiol Lett.

[CR34] Ninnemann H, Maier J (1996). Indications for the occurrence of nitric oxide synthases in fungi and plants and the involvement in photoconidiation of *Neurospora crassa*. Photochem Photobiol.

[CR35] Poyedinok NL, Mykhailova OB, Shcherba VV, Buchalo AS, Negriyko AM (2008). Light regulation of growth and biosynthetic activity of Ling Zhi or Reishi medicinal mushroom, *Ganoderma lucidum* (W. Curt.: Fr.) P. Karst. (Aphyllophoromycetideae), in pure culture. Int J Med Mushrooms.

[CR36] Purschwitz J, Müller S, Kastner C, Schöser M, Haas H, Espeso EA, Atoui A, Calvo AM, Fischer R (2008). Functional and physical interaction of blue- and red-light sensors in *Aspergillus nidulans*. Curr Biol.

[CR37] Röhrig J, Kastner C, Fischer R (2013). Light inhibits spore germination through phytochrome in *Aspergillus nidulans*. Curr Genet.

[CR38] Schumacher J, Gorbushina AA (2020). Light sensing in plant- and rock-associated black fungi. Fungal Biol.

[CR39] Shu CH, Peng JC, Tsai CC (2010). Effects of light intensity and light wavelength on the production of mycophenolic acid by *Penicillium brevicompactum* in batch cultures. Enzyme Microb Technol.

[CR40] Sun CX, Ma YJ, Wang JW (2017). Enhanced production of hypocrellin A by ultrasound stimulation in submerged cultures of *Shiraia bambusicola*. Ultrason Sonochem.

[CR41] Sun CX, Ma YJ, Wang JW (2018). Improved hypocrellin A production in *Shiraia bambusicola* by light-dark shift. J Photochem Photobiol B-Biol.

[CR42] Thevissen K, Terras FRG, Broekaert WF (1999). Permeabilization of fungal membranes by plant defensins inhibits fungal growth. Appl Environ Microbiol.

[CR43] Tisch D, Schmoll M (2010). Light regulation of metabolic pathways in fungi. Appl Microbiol Biotechnol.

[CR44] Tong ZW, Mao LW, Liang HL, Zhang ZB, Wang Y, Yan RM, Zhu D (2017). Simultaneous determination of six perylenequinones in *Shiraia* sp. Slf14 by HPLC. J Liq Chromatogr Relat Technol.

[CR45] Turrion-Gomez JL, Benito EP (2011). Flux of nitric oxide between the necrotrophic pathogen *Botrytis cinerea* and the host plant. Mol Plant Pathol.

[CR46] Verde-Yáñez L, Usall J, Teixidó N, Vall-llaura N, Torres R (2023). Deciphering the effect of light wavelengths in *Monilinia* spp. DHN-melanin production and their interplay with ROS metabolism in *M. fructicola*. J Fungi.

[CR47] Wayne R, Hepler PK (1985). Red light stimulates an increase in intracellular calcium in the spores of *Onoclea sensibilis*. Plant Physiol.

[CR48] Wu SJ, Liu YS, Wu JY (2008). The signaling role of extracellular ATP and its dependence on Ca^2+^ flux in elicitation of *Salvia miltiorrhiza* hairy root cultures. Plant Cell Physiol.

[CR49] Xu MJ, Dong JF, Zhu MY (2005). Effect of nitric oxide on catharanthine production and growth of *Catharanthus roseus* suspension cells. Biotechnol Bioeng.

[CR50] Xu MJ, Dong JF, Zhu MY (2005). Nitric oxide mediates the fungal elicitor-induced hypericin production of *Hypericum perforatum* cell suspension cultures through a jasmonic-acid-dependent signal pathway. Plant Physiol.

[CR51] Xu MJ, Dong JF, Zhu MY (2006). Nitric oxide mediates the fungal elicitor-induced puerarin biosynthesis in *Pueraria thomsonii* Benth. suspension cells through a salicylic acid (SA)-dependent and a jasmonic acid (JA)-dependent signal pathway. Sci China Ser C Life Sci.

[CR52] Xu CL, Lin WX, Chen YN, Gao BL, Zhang ZB, Zhu D (2023). Heat stress enhanced perylenequinones biosynthesis of *Shiraia* sp. Slf14(w) through nitric oxide formation. Appl Microbiol Biotechnol.

[CR53] Yamasaki H, Sakihama Y (2000). Stimultaneous production of nitric oxide and peroxynitrite by plant nitrate reductase: *in vitro* evidence for the NR-dependent formation of active nitrogen species. FEBS Lett.

[CR54] Yang HL, Xiao CX, Ma WX, He GQ (2009). The production of hypocrellin colorants by submerged cultivation of the medicinal fungus *Shiraia bambusicola*. Dyes Pigment.

[CR55] Zhao D, Liang ZQ (2005). Reviews of studies on isolation and culture of *Shiraia bambusicola* Henn. J Fungal Res.

[CR56] Zhao N, Lin X, Qi SS, Luo ZM, Chen SL, Yan SZ (2016). *De novo* transcriptome assembly in *Shiraia bambusicola* to investigate putative genes involved in the biosynthesis of hypocrellin A. Int J Mol Sci.

[CR57] Zhao YX, Lim J, Xu JY, Yu JH, Zheng WF (2020). Nitric oxide as a developmental and metabolic signal in filamentous fungi. Mol Microbiol.

[CR58] Zhao N, Yu YY, Yue YX, Dou MZ, Guo BJ, Yan SZ, Chen SL (2021). Nitric oxide regulates perylenequinones biosynthesis in *Shiraia bambusicola* S4201 induced by hydrogen peroxide. Sci Rep.

[CR59] Zhao YX, Yuan WW, Sun MN, Zhang XG, Zheng WF (2021). Regulatory effects of nitric oxide on reproduction and melanin biosynthesis in onion pathogenic fungus *Stemphylium eturmiunum*. Fungal Biol.

[CR60] Zheng WF, Miao KJ, Zhang YX, Pan SY, Zhang MM, Jiang H (2009). Nitric oxide mediates the fungal-elicitor-enhanced biosynthesis of antioxidant polyphenols in submerged cultures of *Inonotus obliquus*. Microbiology.

[CR61] Zhong JJ, Xiao JH (2009). Secondary metabolites from higher fungi: discovery, bioactivity, and bioproduction. Adv Biochem Eng Biotechnol.

[CR62] Ziv C, Feldman D, Aharoni-Kats L, Chen S, Liu Y, Yarden O (2013). The N-terminal region of the *Neurospora* NDR kinase COT1 regulates morphology via its interactions with MOB2A/B. Mol Microbiol.

