# Supplementary material for: Nitric oxide mediates red light-induced perylenequinone production in Shiraia mycelium culture
Source: Bioresour Bioprocess. 2024 Jan 2;11(1):2. doi: 10.1186/s40643-023-00725-5 (PMC10991179; doi:10.1186/s40643-023-00725-5)
Supplement: Supplementary file 1 — Additional file 1: Table S1. Primers and relevant information of reference and target genes. F: forward primer, R: reverse primer. Fig. S1. HPLC chromatograms of PQ standards. 1. elsinochrome C; 2. elsinochrome B; 3. hypocrellins C; 4. elsinochrome A; 5. hypocrellins A. Fig. S2. Effects of red light on the pH value (A) and residual sugar (B) in Shiraia sp. S9. The culture was maintained in a 150 mL flask containing 50 mL medium at 28℃ and 150 r/min under the dark or red light treatment. The intensity of red light (627 nm) was 200 lx. Values are mean ± SD from three independent experiments. Fig. S3. Effects of different concentrations of SNP under red light treatment on biomass (A), HA content in mycelium (B), the released HA in cultural broth (C) and total HA production (D) of Shiraia sp. S9. The culture was maintained in a 150 mL flask containing 50 mL medium at 150 r/min and 28℃ under the dark or red light treatment for 8 days. The intensity of red light (627 nm) was 200 lx. SNP (1, 5, 10 and 20 μM) were added 30 min prior to the red light treatment. Values are mean ± SD from three independent experiments (*p< 0.05 and **p < 0.01 vs. control. #p < 0.05 and ##p < 0.01 vs. red light treatment). Fig. S4. Effects of addition time of SNP (5 μM) under red light treatment on HA production of Shiraia sp. S9. The fungal dry biomass (A), HA content in mycelium (B), the released HA in cultural broth (C) and total HA production (D) in liquid culture. The culture was maintained in a 150 mL flask containing 50 mL medium at 150 r/min and 28℃ under the dark or red light treatment for 8 days. The intensity of red light (627 nm) was 200 lx. SNP (5 μM) was added on day 1-5 of culture, 30 min prior to the red light treatment. Values are mean ± SD from three independent experiments (*p < 0.05 and **p < 0.01 vs. control. #p < 0.05 and ##p < 0.01 vs. red light treatment). [file 40643_2023_725_MOESM1_ESM.pdf]

Supplementary materials

Nitric oxide mediates red light-induced perylenequinone production in *Shiraia* mycelium culture

Wen Juan Wang, Xin Ping Li, Wen Hao Shen, Qun Yan Huang, Rui Peng Cong
Li Ping Zheng, Jian Wen Wang

✉ Li Ping Zheng, Jian Wen Wang
lpzheng@suda.edu.cn; jwwang@suda.edu.cn

Table S1. Primers and relevant information of reference and target genes. F: forward primer, R: reverse primer.

Fig. S1. HPLC chromatograms of PQ standards. 1. Elsinochrome C; 2. Elsinochrome B; 3. Hypocrellin C; 4. Elsinochrome A; 5. Hypocrellins A.

Fig. S2. Effects of red light on the pH value (A) and residual sugar (B) in *Shiraia* sp. S9. The culture was maintained in a 150 mL flask containing 50 mL medium at 28°C and 150 r/min under the dark or red light treatment. The intensity of red light (627 nm) was 200 lx. Values are mean ± SD from three independent experiments.

Fig. S3. Effects of different concentrations of SNP under red light treatment on biomass (A), HA content in mycelium (B), the released HA in cultural broth (C) and total HA production (D) of *Shiraia* sp. S9. The culture was maintained in a 150 mL flask containing 50 mL medium at 150 r/min and 28°C under the dark or red light treatment for 8 days. The intensity of red light (627 nm) was 200 lx. SNP (1, 5, 10 and 20 μM) were added 30 min prior to the red light treatment. Values are mean ± SD from three independent experiments ($^{*}p < 0.05$ and $^{**}p < 0.01$ vs. control. $^{\#}p < 0.05$ and $^{\#\#}p < 0.01$ vs. red light treatment).

Fig. S4. Effects of addition time of SNP (5 μM) under red light treatment on HA production of *Shiraia* sp. S9. The fungal dry biomass (A), HA content in mycelium (B), the released HA in cultural broth (C) and total HA production (D) in liquid culture. The culture was maintained in a 150 mL flask containing 50 mL medium at 150 r/min and 28°C under the dark or red light treatment for 8 days. The intensity of red light (627 nm) was 200 lx. SNP (5 μM) was added on day 1-5 of culture, 30 min prior to the red light treatment. Values are mean ± SD from three independent experiments ($^{*}p < 0.05$ and $^{**}p < 0.01$ vs. control. $^{\#}p < 0.05$ and $^{\#\#}p < 0.01$ vs. red light treatment).

Table S1

Primers and relevant information of reference and target genes. F: forward primer, R: reverse primer.

Gene symbol	Gene name	Sequence
18S	18S rRNA gene	F: ACGCAGCGAAATGCGATAAG<br>R: CAAATTGTGCTGCGCTCCAA
PKS	Polyketide synthase	F: TGCTGAGGTAGCAGTCAAGC<br>R: TTATGCTACGGTCGTCGCTC
Omef	*O*-methyltransferase	F: GAACTACCTGAAGGCACGCT<br>R: GCTCGGAAGGATACTCGCTC
Mono	Salicylate 1-monooxygenase	F: TCTCGGGGAATTATGGCACG<br>R: ACAACCGTTCTCGCATCAGT
FAD	FAD/FMN-containing dehydrogenase	F: TGTGACCGCCATCACCTTAC<br>R: TTGTCGTATGGGTGGGAAGC
MCO	Multicopper oxidase	F: TATGGCGCTACGAGTGGAC<br>R: ACTCCCTGGCCGATAACGTA
ZFTF	Zinc finger transcription factor	F: GAACACCGTCGCAAGATTCG<br>R: TCATTGGCATCGCTTGGAGT
MFS	Major facilitator superfamily	F: TCCCGTAGCCTTGCTTTCTG<br>R: CCGGCTTCTTCTTGACGCTA
SSPK	stage v sporulation protein k	F: ATGAGAAGGAGCGGCGTATG<br>R: GTATGACTTCCACCCGCACA
ISP7	sexual differentiation process protein	F: GCCAGCAGTTCAGGGATACA<br>R: AAGATGGTGATGGCACCGAA
CSN3	cleistothecium development	F: CTAGATACGCCATGCCTCCC<br>R: TACGTCTCGACCCTACCACA
BrlA	transcriptional factor	F: GTCCAGACATTCACCCCCAG<br>R: CTTGTTGACATTGGGCTGGC
WetA	transcriptional factor	F: CACCTCGACCTCACACTTCC<br>R: GGATGGCGATGAGACTGGAG
HSP30	30 kDa heat shock protein	F: GTCGTCGCAGCACATCTCAA<br>R: TCCGGCTTCGTTCTTACAGC

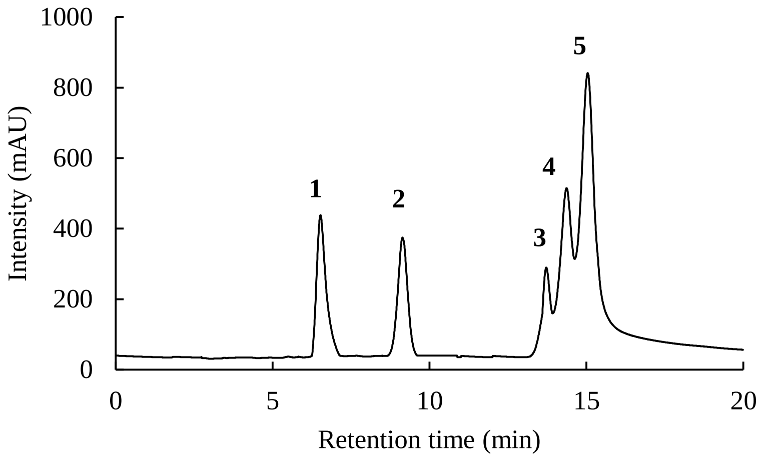

Fig. S1. HPLC chromatograms of PQ standards. 1. Elsinochrome C; 2. Elsinochrome B; 3. Hypocrellin C; 4. Elsinochrome A; 5. Hypocrellins A.

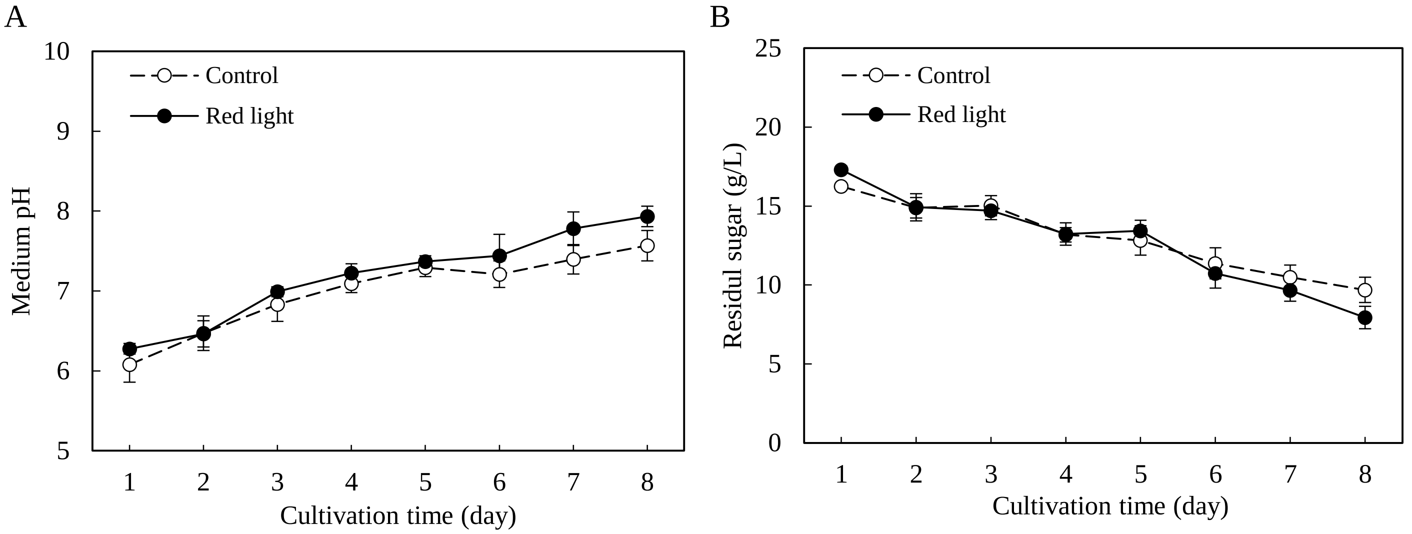

Fig. S2. Effects of red light on the pH value (A) and residual sugar (B) in *Shiraia* sp. S9. The culture was maintained in a 150 mL flask containing 50 mL medium at 28°C and 150 r/min under the dark or red light treatment. The intensity of red light (627 nm) was 200 lx. Values are mean ± SD from three independent experiments.

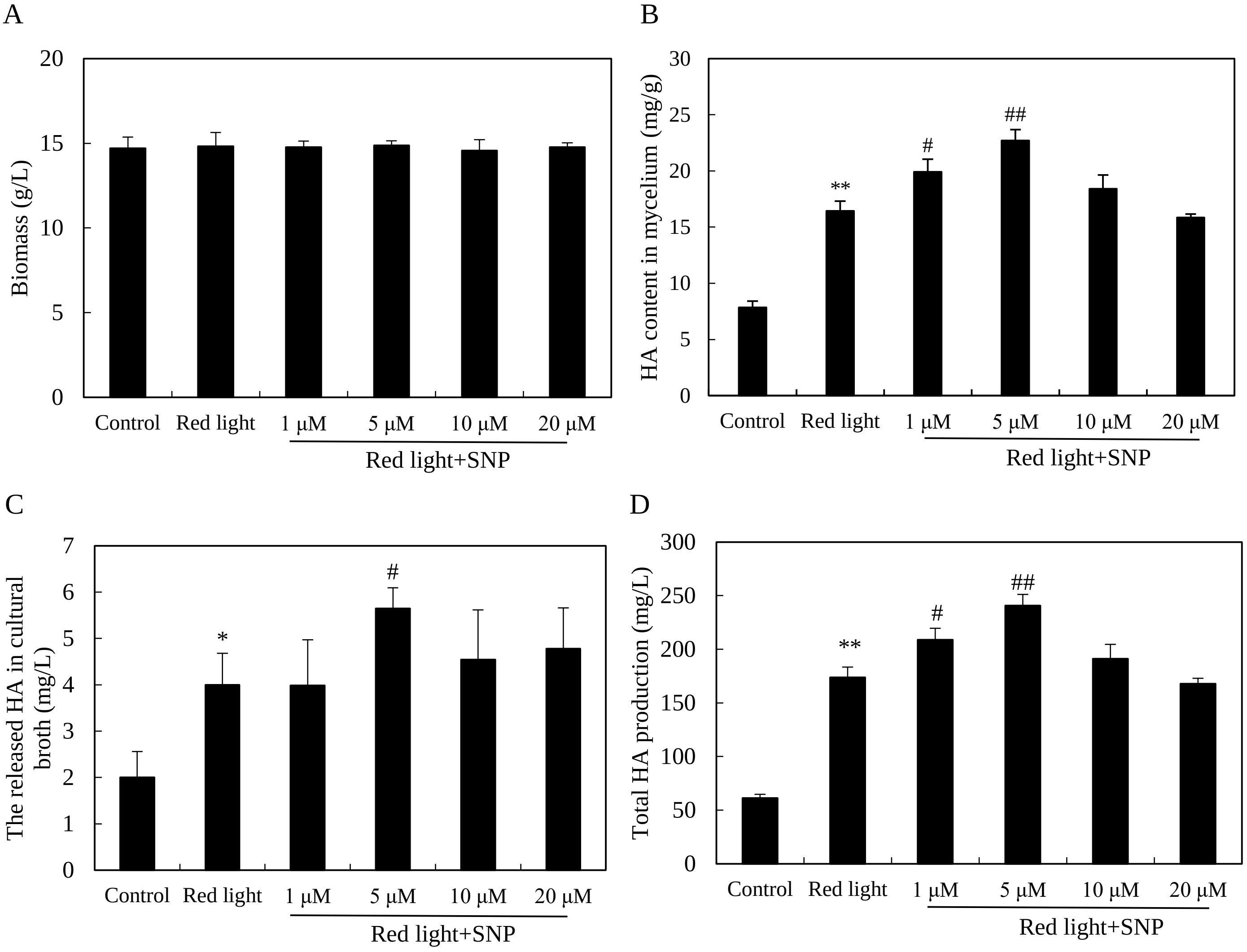

Fig. S3. Effects of different concentrations of SNP under red light treatmet on biomass (A), HA content in mycelium (B), the released HA in cultural broth (C) and total HA production (D) of *Shiraia* sp. S9. The culture was maintained in a 150 mL flask containing 50 mL medium at 150 r/min and 28℃ under the dark or red light treatment for 8 days. The intensity of red light (627 nm) was 200 lx. SNP (1, 5, 10 and 20 μM) were added 30 min prior to the red light treatment. Values are mean ± SD from three independent experiments (*$p < 0.05$ and **$p < 0.01$ vs. control. $^{\#}p < 0.05$ and $^{\#\#}p < 0.01$ vs. red light treatment).

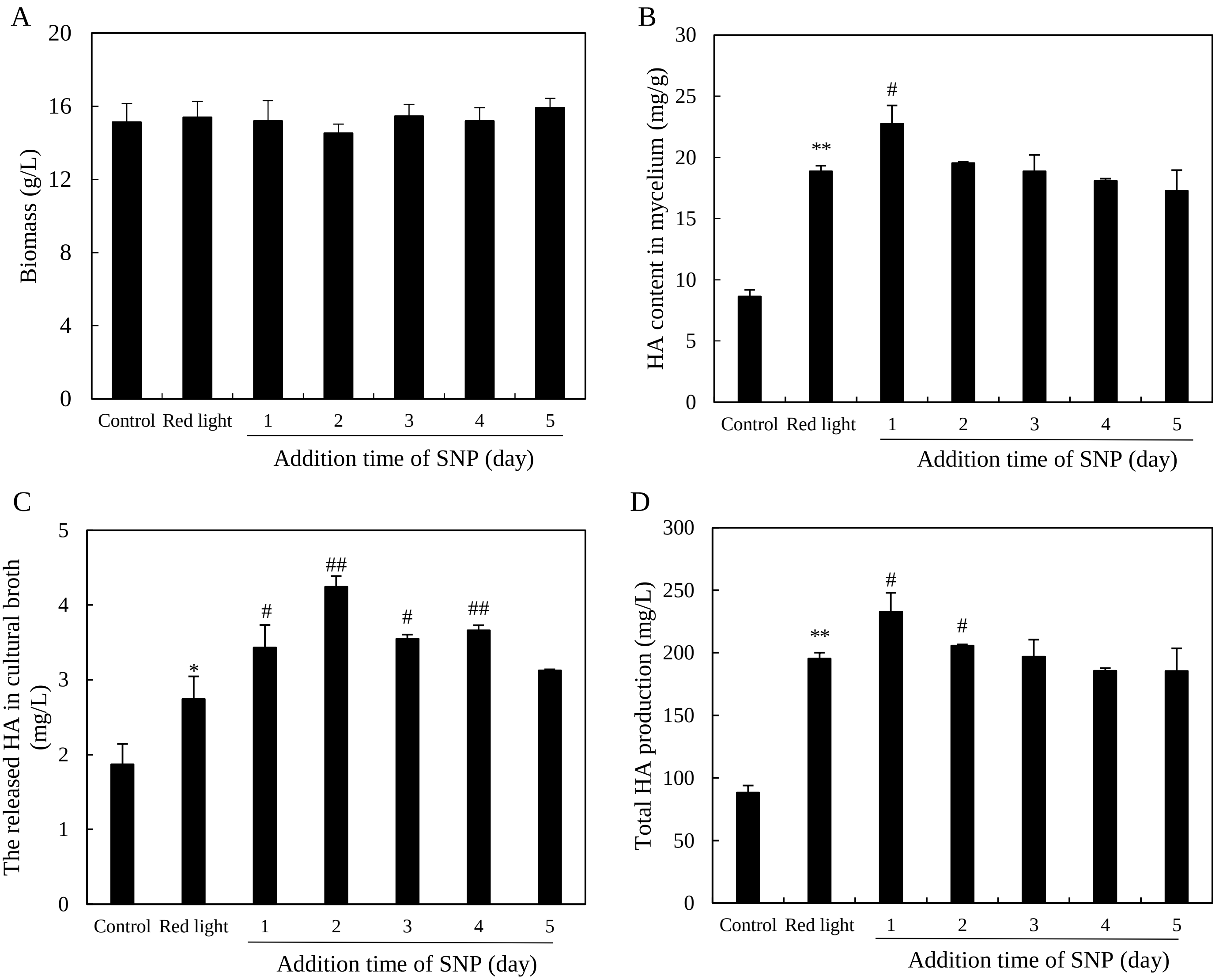

Fig. S4. Effects of addition time of SNP (5 μM) under red light treatment on HA production of *Shiraia* sp. S9. The fungal dry biomass (A), HA content in mycelium (B), the released HA in cultural broth (C) and total HA production (D) in liquid culture. The culture was maintained in a 150 mL flask containing 50 mL medium at 150 r/min and 28°C under the dark or red light treatment for 8 days. The intensity of red light (627 nm) was 200 lx. SNP (5 μM) was added on day 1-5 of culture, 30 min prior to the red light treatment. Values are mean ± SD from three independent experiments (*p< 0.05 and **p < 0.01 vs. control; $^{\#}p$ < 0.05 and $^{\#\#}p$ < 0.01 vs. red light treatment).
